# XJB-5-131-mediated improvement in physiology and behaviour of the *R6/2* mouse model of Huntington's disease is age- and sex- dependent

**DOI:** 10.1371/journal.pone.0194580

**Published:** 2018-04-09

**Authors:** Aris A. Polyzos, Nigel I. Wood, Paul Williams, Peter Wipf, A. Jennifer Morton, Cynthia T. McMurray

**Affiliations:** 1 Molecular Biophysics and Integrated Bioimaging Division, Lawrence Berkeley National Laboratory, Berkeley, CA, United States of America; 2 Department of Physiology, Development, and Neuroscience, Anatomy Building, University of Cambridge, Cambridge, United Kingdom; 3 Department of Chemistry, University of Pittsburgh, Pittsburgh, PA, United States of America; Max Delbruck Centrum fur Molekulare Medizin Berlin Buch, GERMANY

## Abstract

We have reported that the radical scavenger XJB-5-131 attenuates or reverses progression of the disease phenotype in the *HdhQ(150/150)* mouse, a slow onset model of HD. Here, we tested whether XJB-5-131 has beneficial effects in *R6/2* mice, a severe early onset model of HD. We found that XJB-5-131 has beneficial effects in *R6/2* mice, by delaying features of the motor and histological phenotype. The impact was sex-dependent, with a stronger effect in male mice. XJB-5-131 treatment improved some locomotor deficits in female *R6/2* mice, but the effects were, in general, greater in male mice. Chronic treatment of male *R6/2* mice with XJB-5-1-131 reduced weight loss, and improved the motor and temperature regulation deficits, especially in male mice. Treatment with XJB-5-131 had no effect on the lifespan of *R6/2* mice. Nevertheless, it significantly slowed somatic expansion at 90 days, and reduced the density of inclusions. Our data show that while treatment with XJB-5-131 had complex effects on the phenotype of *R6/2* mice, it produced a number of significant improvements in this severe model of HD.

## Introduction

**Fig 1:**
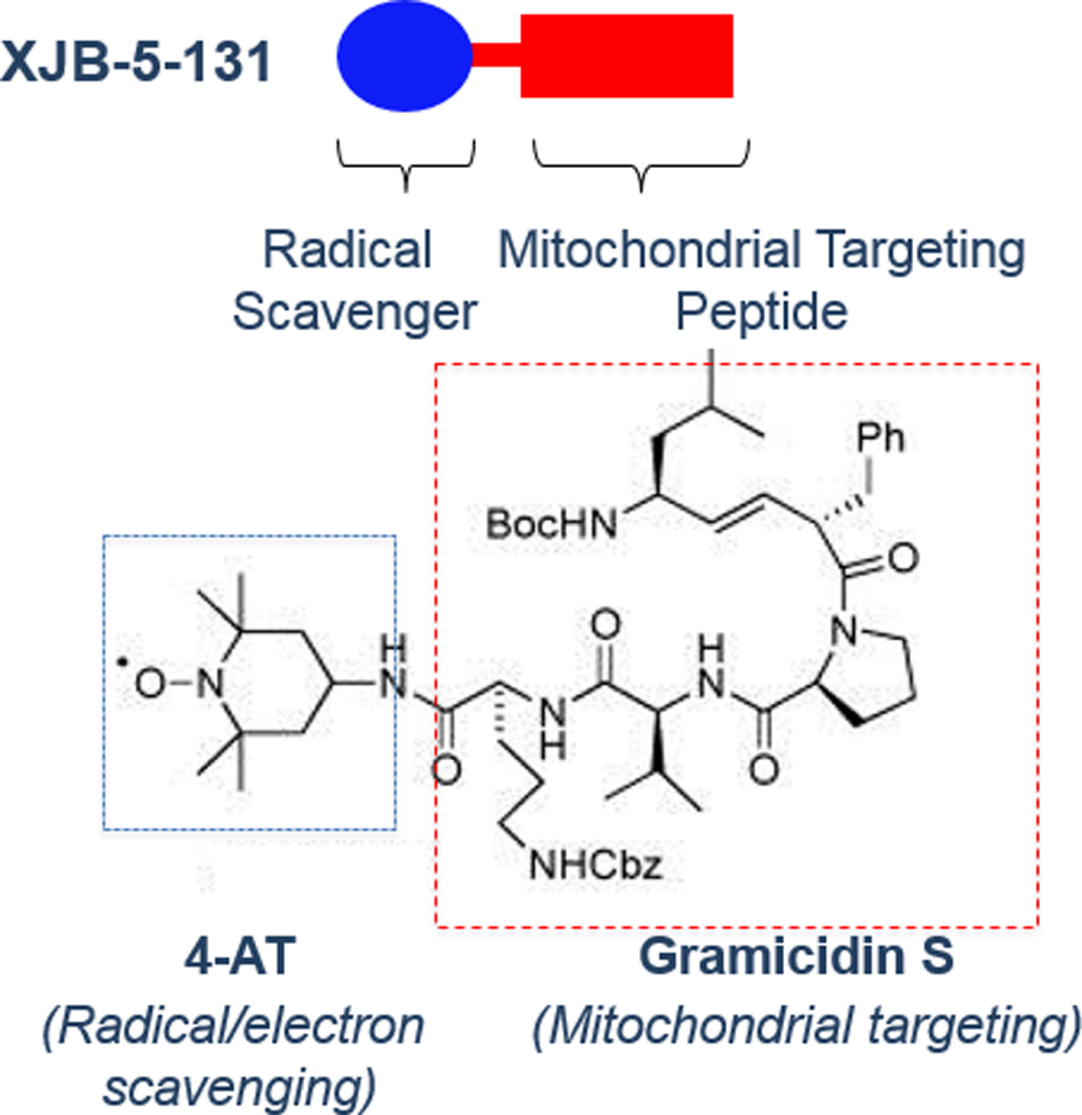
The structure of XJB-5-131. XJB-5-131 is a bi-functional antioxidant comprising a delivery component conjugated to an antioxidant moiety [1, 4]. The delivery portion of the molecule is an alkene peptide isostere modification of the Leu-D-Phe-Pro-Val-Orn segment of the antibiotic gramicidin S (red) [15]. This peptide mimetic directly targets the mitochondrial membrane and delivers the antioxidant nitroxide, 2,2,6,6-tetramethyl piperidine-1-oxyl (TEMPO) (blue), to neutralize reactive radical species.

XJB-5-131 is a bi-functional synthetic antioxidant comprising a delivery component conjugated to an antioxidant moiety (Fig 1)[1–4]. This peptide mimetic portion of XJB-5-131 (Fig 1, red) directly targets the mitochondrial membrane and delivers the antioxidant nitroxide (Fig 1, blue) to neutralize reactive radical species [5]. XJB-5-131 has profound beneficial effects in offsetting the effects of oxidative damage [2, 6–9]. We have previously reported the effects of XJB-5-131 in the *HdhQ(150/150)* mouse model of Huntington’s disease [8–10], which carries a disease-length 150 CAG tract knocked into both full-length endogenous alleles [11]. These mice develop pathophysiology slowly. *HdhQ(150/150)* animals typically live as long as their wild-type counterparts, but develop features of disease at approximately 20–25 weeks for homozygotic mice and upwards of 60 weeks for heterozygotic mice [11–14]. When treatment was started before the phenotype developed (7 weeks of age), XJB-5-131 attenuated the decline in rotarod performance, suppressed weight loss, and increased the copy number of mitochondrial DNA in *HdhQ(150/150)* mice [9]. XJB-5-131 also attenuated decline or reversed the effects of disease if treatment began after disease onset at 60 weeks of age [8]. In ageing animals with well-developed pathology, XJB-5-131 treatment promoted weight gain, attenuated neuronal loss, reduced inclusion formation, prevented decline in performance in multiple motor tests, and decreased oxidative damage in the brains of aging *HdhQ(150/150)* animals relative to age-matched vehicle-treated *HdhQ(150/150)* mice [8]. XJB-5-131 also reduced the level of 8-oxo-guanine and of cardiolipin oxidation in treated mice [6, 8, 15]. Cardiolipin is an important component of the inner mitochondrial membrane, and is essential for the proper functioning of numerous enzymes and optimal mitochondrial energy metabolism [16].

In contrast to full-length mouse models, fragment HD model mice express only an N-terminal polyglutamine portion of the HD disease gene. The most commonly used of these models is the *R6/2* line [17–20]. The *R6/2* mouse phenotype is particularly acute in the line with ~140 repeats, presenting with overt symptoms at 9–11 weeks and dying by 13–18 weeks [17]. The CAG repeat tract in the 5’ exon 1 coding sequence of both *R6/2* and the related *R6/1* lines of HD mouse is unstable [19, 21–25]. While the transgene original founder retained a CAG tract of ~144 repeats [16, 19], mice with repeats longer than 350 develop phenotypes later, and survive longer, than those with shorter repeats [21, 26, 27].

Because *R6/2* mice develop a severe, progressive behavioural phenotype that mimics early onset aspects of HD pathophysiology [17–20], we were interested in finding out if XJB-5-131 could ameliorate phenotypic signs of HD in *R6/2* mice. The *R6/2* mice were tested for changes in overall physiology and behaviour in a series of behavioural tests. Here, we report that XJB-5-131 has beneficial effects in *R6/2* mice. Consistent with its role as an oxygen radical scavenger, XJB-5-131 suppressed age-dependent somatic CAG expansion in both male and female *R6/2* mice. The impact of XJB-5-131 on the outcome of behavioural testing was sex-dependent, with male mice showing greater improvement.

## Materials and methods

### Mice

This research was regulated under the Animals (Scientific Procedures) Act 1986 Amendment Regulations 2012, and following ethical review and approval by the University of Cambridge Animal Welfare and Ethical Review Body. Mice were taken from colonies of *R6/2* mice established in the University of Cambridge, and maintained by backcrossing onto *CBA* x *C57BL6* F1 female mice. Genotyping methods and detailed husbandry for *R6/2* animals have been described previously [20, 26]. Briefly, mice were housed in single-sex, single-genotype groups of 8–10 at 21–23°C with humidity of 55 ± 10%. Lowered waterspouts were provided for *ad libitum* access to water and standard dry laboratory food was given. A supplementary feed (of mash made by soaking 100g dry food in 230 ml of tap water until pellets were fully expanded) was given each morning and evening. Enrichment was provided by the addition of plastic houses, cardboard tubes and chew blocks to the cages. Mice lived under a 12hr/12hr light/dark cycle, with lights on at 7am and off at 7pm. Genotyping and CAG repeat length measurement were carried out by Laragen (Los Angeles, CA, USA). CAG repeat lengths of the transgenic mice were 255 ± 1 (mean ± SEM), as determined by GeneMapper. Mice were divided into 6 groups: *R6/2* male drug-treated (n = 7) (hereafter referred to as *R6/2*-XJB male); *R6/2* female drug-treated (n = 9) (*R6/2*-XJB female); *R6/2* male vehicle-treated (n = 8) (*R6/2*-Veh male); *R6/2* female vehicle-treated (n = 8) (*R6/2*-Veh female); WT male vehicle-treated (n = 8) (WT-Veh male); and WT female vehicle-treated (n = 8) (WT-Veh female).

### XJB-5-131 preparation

XJB-5-131 was synthesized as previously reported [8, 9]. XJB-5-131 was stored as powder at -80°C. At the beginning of the experiment, lyophilized, powdered XJB-5-131 was reconstituted in DMSO at a concentration of 1 mg/μL, as previously described [8]. On each dosing day, the XJB-5-131 solution was mixed with filtered and pre-heated PBS (100°C), and heated for 10 seconds. The final concentration was 2mg/kg mouse body weight in a 200 μL volume. The solution was injected within 30 minutes of preparation.

### Drug treatment

WT or *R6/2* animals were treated with either XJB-5-131 (XJB) or the saline vehicle (Veh). We have previously published that XJB-5-131 has little effects on WT animals [8]. Thus, we focused here on the impact of XJB-5-131 on improving the phenotype of R6/2 mice, using WT-Veh as a control. XJB-5-131 or vehicle was administered intraperitoneally every second day. Dosing was carried out in the evening. On days where behavioural testing was being conducted, dosing was done after testing to avoid detecting acute drug effects. XJB-5-131 and vehicle were aliquoted into coded bottles to avoid bias. Drug treatment began at 4 weeks of age.

### Behavioural assessments

**Fig 2:**
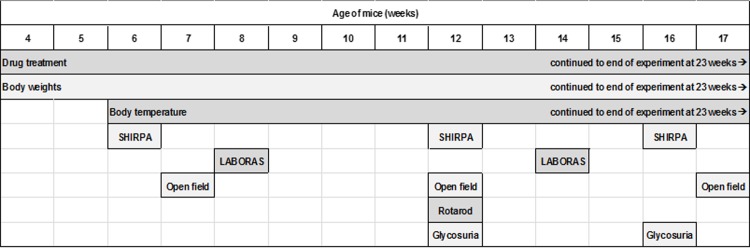
Timeline of behavioural study.

All behavioural testing was performed at the same time of day by the same experimenter. At the beginning of the experiment, all behavioural testing was carried out blind to genotype. As the phenotype became apparent, this was no longer possible. A timeline of the behavioural assessments is shown (Fig 2).

### Body temperatures

Implantable electronic transponders (IPTT300, Bio Medic Data Systems Inc., Seaford, DE) were injected subcutaneously between the scapulae of mice at 6 weeks of age. Temperatures were recorded every second day before drug/vehicle administration, and averaged over each week, to eliminate the confounding effect of day-to-day variations in individual mice.

### Body weights

Body weights were recorded every second day from 4 weeks of age. Weights were taken before drug/vehicle administration at the same time as temperature recording, and averaged over each week, to eliminate the confounding effect of day-to-day variations in individual mice.

### Accelerating rotarod

Mice were tested once on the accelerating rotarod (Ugo Basile, Varese, Italy) task at 12 weeks of age. Each mouse received 2 training trials per day for 4 days. Each training trial consisted of placing the mouse on the rod rotating at a speed of 24 rpm for 60s. For the test itself, mice were placed on the rotarod, which was accelerated from 4–40 rpm over a 10 minute period. The latency to fall or step off the rotarod was calculated as an average of 3 trials. Inter-trial intervals were 45–50 min for each mouse.

### Fixed speed rotarod

Mice were tested on the fixed speed rotarod on the day after completing the accelerating rotarod procedure. Each mouse was given 2 successive trials at each of 7 different speeds (5, 8, 15, 20, 24, 31, 33 and 40 rpm). At each speed, the latency to fall off the rotarod (maximum 60s) was recorded, and the average of both trials at each speed was used for the analysis. Inter-trial intervals were 5–10 min for each mouse.

### Open field test

Mice were evaluated in the open-field task as described by Carter et al. [17]. Briefly, mice were placed individually in an open-topped plastic box 60 × 60 × 30 cm high, with white walls and floor. The floor was marked with black gridlines (divided into 25 squares (‘cells’), each measuring 12 × 12 cm). The middle nine cells were called the centre, the outer ‘ring’ of cells was called the periphery. Individual mice were placed in the central cell of the open field and observed for a 10 minute period. Parameters measured included: (1) latency to reach the periphery; (2) total number of central cells entered (defined as three or more paws moving into a central cell); (3) total number of peripheral cells entered (defined as three or more paws moving into a peripheral cell); (4) total number of cells entered; (5) total incidences of supported rearing (standing up on hind legs using the wall for support); (6) total incidences of unsupported rearing (standing up on hind legs without wall support); (7) number of complete grooming cycles; (8) number of faecal boli; and (9) number of urinations. Mice were tested at 7, 12 and 17 weeks.

### LABORAS

Normal mouse behaviours were measured over 48 hours in the LABORAS (Laboratory Animal Behaviour, Observation, Registration and Analysis System) apparatus (Metris b.v., Hoofddorp, The Netherlands). The LABORAS apparatus measures locomotor activity, immobility, grooming, eating, drinking and climbing. During the monitoring period mice were singly housed with *ad libitum* access to food and water. For each mouse, testing was carried out twice, at 8 and 14 weeks of age. Following testing, the mice were returned to their home cages.

### SHIRPA assessment and hangwire score

The general physical condition of the mice was assessed at 6, 12 and 16 weeks using a modified SHIRPA (SmithKline Beecham Pharmaceuticals; Harwell, MRC Mouse Genome Centre and Mammalian Genetics Unit; Imperial College School of Medicine at St Mary's; Royal London Hospital, St Bartholomew's and the Royal London School of Medicine Phenotype Assessment) protocol [28, 29]. Briefly, mice were assessed for hair and tail morphology, piloerection, presence/absence of whiskers, domed face and lordokyphosis, respiration rate, palpebral closure, color of eye fur, fluidity of gait, pelvic elevation, tail elevation, touch escape, positional passivity, forelimb and hindlimb clasping, aggression, vocalisation, tremor, grip strength, initial activity and righting reflex. Data were quantified using a graded scoring system, where normal behaviour received a score of 0. A global score was determined for each mouse, with the highest scores corresponding to the greatest degree of abnormality. Grip strength was measured as part of the SHIRPA screen, using the hangwire test. Individual mice were placed on a wire cage lid and the lid was gently moved back and forth until the mouse gripped the wire. The lid was then turned upside down, 15cm above the surface of the bedding material. Latency to fall onto the bedding was recorded, with a 60s cut-off time.

### Glycosuria

Mice were placed in a clean, sawdust-free cage. Most mice urinated spontaneously, but where necessary, gentle handling was used to induce micturition. The urine was tested with Diastix reagent strips (Bayer plc, Newbury, UK), which enables a semi-quantitative analysis of glycosuria. The presence of glycosuria was regarded as indicative of diabetic status. Mice were tested at 12 and 16 weeks of age.

### Somatic expansion

The size distribution of CAG repeats was established using GeneMapper [30] and the significance was determined by the method previously reported [10]. The age-dependent somatic changes among treated and untreated groups were normalized by subtracting the CAG tracts measured at birth from the CAG tracts measured at 12 weeks. The distributions were expressed as the change in repeat length and summed from all animals within a treatment group to create a single global distribution that characterized the population. Using these global distributions, somatic expansions in the striatum and cerebellum were a measure of the overall changes in repeat length in treated or untreated groups. The distributions at 12 weeks were divided into 100 cells (quantile analysis), excluding the first and last 5 quantiles, and the length of peaks in each cell was averaged, and the difference in the means of treated and untreated animals were subtracted. Data were expressed as a comparison between treated versus the untreated. The means between groups were performed using one-way analysis of variance (ANOVA) with the categories of treatment or age as independent factors. For analyses of means involving the treated and untreated groups, the F-test was used to determine whether the variances between the two groups were significantly different. For samples with a significant difference in variance, the Welch’s t-test was applied. Student’s t-test was applied for the samples with an insignificant difference in variance. The significance level was set at 0.05 for all analyses. All statistical analysis computations were carried out using Prism (Graphpad Software).

### Survival

Age of death was recorded for all *R6/2* mice. Mice were killed when they reached end-point—if they were moribund, lacked a righting reflex, or failed to respond to gentle stimulation.

### Tissue analysis

Subsets of mice (n = 2 per group) were killed for histology at 8 and 12 weeks.

### Histology

Primary antibodies used were mouse anti-NeuN Alexa488 conjugate (Millipore #MAB377X) (used at 1:400), mouse anti-GFAP Cy3 conjugate (Abcam #ab49874) (used at 1:400, and rabbit anti-Ubiquitin (DAKO #Z0458) (used at 1:400). Secondary antibodies used were goat anti-rabbit Alexa-555 conjugated (Invitrogen #A31630) (used at 1:400), goat anti-mouse Alexa-488 conjugated (Invitrogen #A31620) (used at 1:400). Brain sections cryoembedded in OCT were sliced (10 μm thick using a Leica Cryostat set at: -14°C for the sample and -12°C for the blade) and placed onto Histobond microscope slides (VWR). They were immediately fixed and OCT removed in 100% methanol (10min). Samples were rehydrated sequentially in 75%, 50%, 25% and 0% ethanol in PBS (2min each). Tissue was treated with Image-iT FX signal enhancer (ThermoFisher #I36933) to reduce autofluorescence (30min) and blocked (2-18hrs) in blocking solution (PBS, 3% BSA, 5% goat serum, 0.7% donkey serum, 0.03% triton X-100). Antibody staining was performed overnight, followed by 3 washes with PBS (5min ea.). Secondary antibody was applied later as required along with 0.5 μM DAPI (1-2hrs) followed by 3 washes in PBS. Slides were coated with Vectashield+DAPI, sealed with a coverslip and stored (-20°C) until they were imaged. Slides were imaged using a Zeiss 710 confocal microscope using either of 20x(0.8N/A)/air, 40x(1.2N/A)/water or 100x(1.4N/A)/oil lenses. Image analysis was carried out using ImageJ:Fiji [31].

### GeneMapper analysis of somatic CAG expansion

DNA was prepared from frozen mouse tissues and CAG repeat sizing performed by Laragen (Culver City, CA, U.S.A.), as previously reported [23].

### Statistics for behaviour tests

Statistical analyses were performed using Statsoft Statistica v11 software (Statsoft, Tulsa, OK, USA) or Prism 5 (GraphPad Software Inc., San Diego, CA, USA). For statistical analysis of behavioural testing, body weights and temperatures, we used repeated measures ANOVA with sex, treatment and genotype as factors. Bonferroni's *post hoc* test was used to determine specific differences, when significant group effects were found. Survival data were compared using the log-rank test. Significance levels were set at p<0.05 for all analyses. Data were not collated and analysed until the end of the experiment. Behavioural testing was performed in the University of Cambridge, while the somatic expansion analysis and tissue analysis was performed in Lawrence Berkeley Laboratory. The results were shared upon completion of analyses in both laboratories.

## Results

### XJB-5-131 treatment reduces loss of body temperature in *R6/2* mice

**Fig 3:**
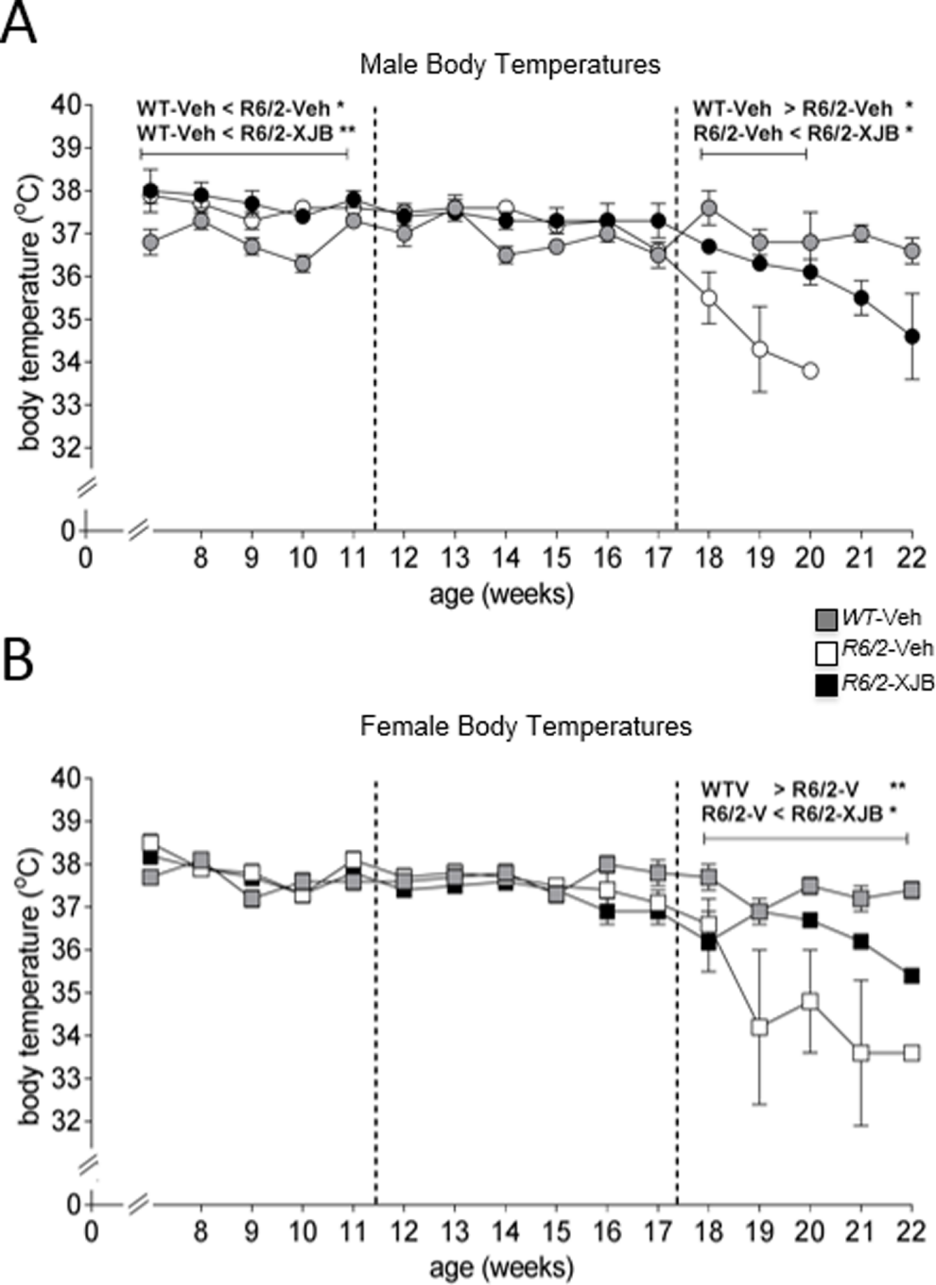
XJB-5-131 suppressed the decline in body temperatures in *R6/2* mice. Body temperatures were recorded every 2 days from implanted microchips. Temperatures were then averaged per week. Panel A, male mice only; B, female mice only. *R6/2* mice showed the expected drop in temperatures with age. Treatment with XJB-5-131 maintained temperatures in *R6/2* mice for longer. * p<0.05, ** p<0.01, *** p<0.001. Grey symbols, WT; white symbols, *R6/2*-Veh; black symbols, *R6/2*-XJB. Dotted lines indicate the analysis periods. Data are mean ± SEM. Mouse numbers: 4–8 weeks, WT male, WT female, *R6/2*-Veh male, *R6/2*-Veh female, n = 8. *R6/2*-XJB male, n = 7; *R6/2*-XJB female, n = 9. 9–12 weeks, all groups n = 6. 13–17 weeks, all groups n = 4. 18 weeks, all groups except *R6/2*-Veh male, n = 4; *R6/2*-Veh male, n = 3. 19 weeks, WT male, WT female, *R6/2*-XJB male, n = 4; *R6/2*-XJB female, n = 3; *R6/2*-Veh male, n = 1; *R6/2*-Veh female, n = 2. 20 weeks, WT male, WT female, n = 4; *R6/2*-XJB male, *R6/2*-Veh female, n = 4; *R6/2*-XJB female, n = 1; *R6/2*-Veh male, n = 0. 21 weeks, WT male, WT female, n = 4; *R6/2*-XJB male, n = 2; *R6/2*-Veh female, *R6/2*-XJB female, n = 1. 22 weeks, WT male, WT female, n = 4; *R6/2*-XJB male, *R6/2*-XJB female, n = 1.

Analysis of body temperature data suggested that there were three distinct phases of disease in the mice. In the early stages of the study (7–11 weeks of age), the body temperature of *R6/2* male mice was higher than WT mice (Fig 3A**),** suggesting a higher resting metabolic rate (WT-Veh vs *R6/2*-Veh, p<0.05; WT vs *R6/2*-XJB, p<0.01). There was no effect of XJB-5-131 treatment (*R6/2*-Veh vs *R6/2*-XJB, p>0.05). Between 12–17 weeks of age, *R6/2* male mice no longer had a higher body temperature than WT male mice (all group comparisons, p>0.05). As the phenotype became more pronounced from 18 weeks onwards, the body temperatures of *R6/2* mice decreased (Fig 3A). Treatment with XJB-5-131 suppressed the drop in temperature (p<0.05).

Female mice showed no genotype or treatment effect on body temperatures at either 7–11 or 12–17 weeks (all comparisons, p>0.05). As seen with the male mice, body temperatures of *R6/2* female mice decreased after 18 weeks, when the phenotype was evident (Fig 3B). Between 18–22 weeks, XJB-5-131 treatment reduced the fall in temperature in *R6/2* mice (p<0.05). *R6/2*-Veh mice were colder than WT and *R6/2*-XJB mice (both comparisons, p<0.05, (Fig 3B), with no difference in body temperature between WT and *R6/2*-XJB mice.

### XJB-5-131-treated male *R6/2* mice maintain weight better than their vehicle-treated littermates

**Fig 4:**
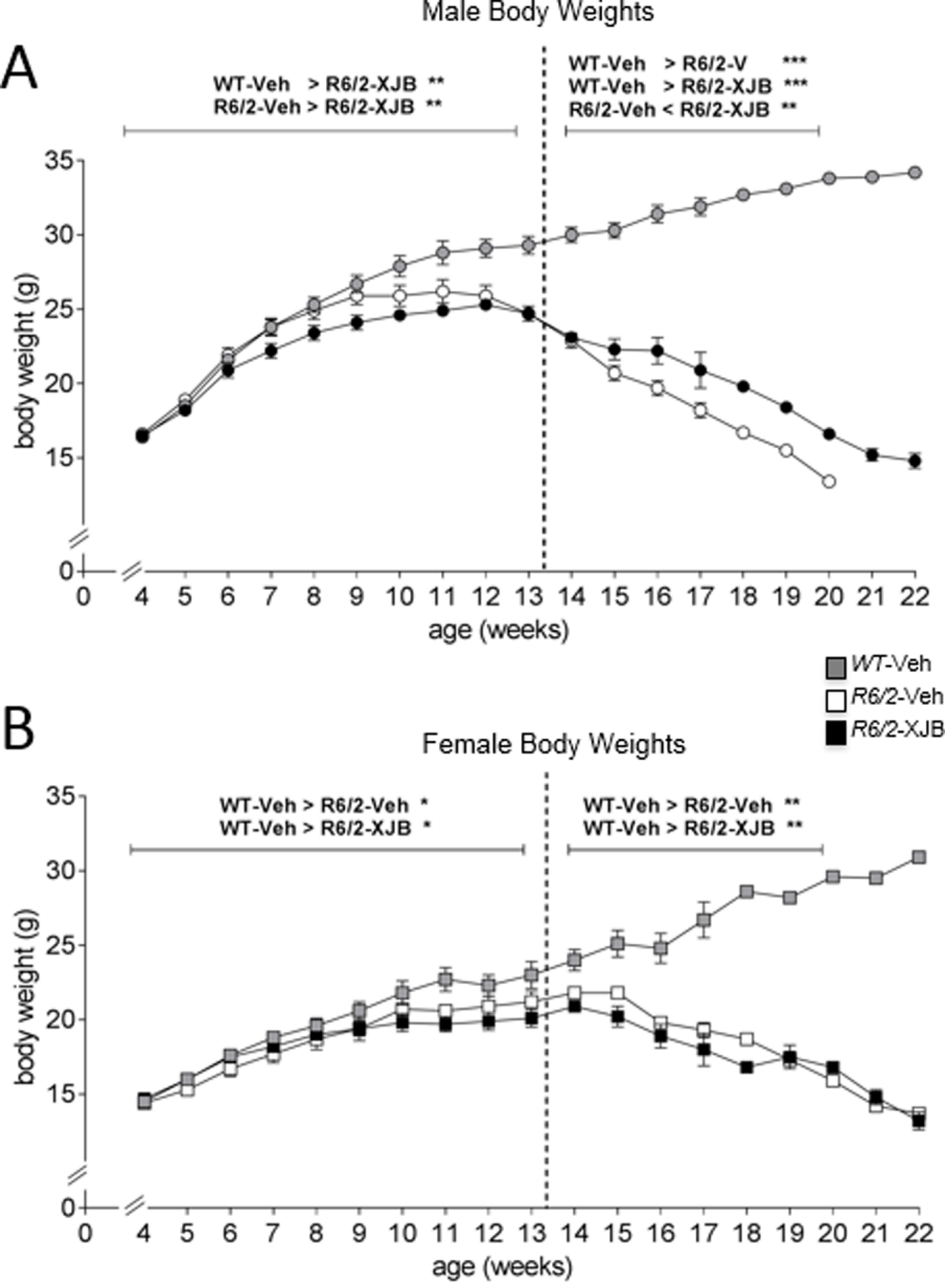
Body weights were recorded every 2 days, and averaged per week. Panel A, male mice only; B, female mice only. *R6/2* mice showed the expected reduction in body weight with age. Treatment with XJB-5-131 caused a slight improvement in maintenance of weight in male (A), but had a detrimental effect in female (B) *R6/2* mice. * p<0.05, ** p<0.01, *** p<0.001. Grey symbols, WT; white symbols, *R6/2*-Veh; black symbols, *R6/2*-XJB. Dotted lines indicate the analysis periods. Data are mean ± SEM. Mouse numbers: 4–8 weeks, WT male, WT female, *R6/2*-Veh male, *R6/2*-Veh female, n = 8. *R6/2*-XJB male, n = 7; *R6/2*-XJB female, n = 9. 9–12 weeks, all groups n = 6. 13–17 weeks, all groups n = 4. 18 weeks, all groups except *R6/2*-Veh male, n = 4; *R6/2*-Veh male, n = 3. 19 weeks, WT male, WT female, *R6/2*-XJB male, n = 4; *R6/2*-XJB female, n = 3; *R6/2*-Veh male, n = 1; *R6/2*-Veh female, n = 2. 20 weeks, WT male, WT female, n = 4; *R6/2*-XJB male, *R6/2*-Veh female, n = 4; *R6/2*-XJB female, n = 1; *R6/2*-Veh male, n = 0. 21 weeks, WT male, WT female, n = 4; *R6/2*-XJB male, n = 2; *R6/2*-Veh female, *R6/2*-XJB female, n = 1. 22 weeks, WT male, WT female, n = 4; *R6/2*-XJB male, *R6/2*-XJB female, n = 1.

Weight loss is a feature of both human and mouse HD pathology. We predicted that if XJB-5-131 treatment was beneficial, it should prevent or slow weight loss in *R6/2* mice. At 4 weeks of age, WT and *R6/2* male mice were of a similar weight, but from this age onward *R6/2* mice, as a consequence of the developing phenotype, failed to gain weight at the same rate as their WT-V littermates (Fig 4). In male mice, there was a biphasic response to drug treatment, with the tipping point at 13 weeks. Between 4 and 13 weeks of age, XJB-5-131-treated *R6/2* male mice failed to gain weight as quickly as either WT or *R6/2*-Veh mice (both comparisons, p<0.01; (Fig 4A). However, from 14 weeks of age onwards, although *R6/2*-XJB male mice continued to lose weight, they did so at a slower rate than *R6/2*-Veh male mice. Female *R6/2* mice also displayed a phenotype-induced weight loss, but progression was slower than in male mice (Fig 4B). Between 4 and 13 weeks of age, all *R6/2* female mice, regardless of treatment, failed to gain weight as quickly as WT mice. Unlike in male mice, however, there was no ameliorating drug effect at later ages, and both drug- and vehicle- treated groups of *R6/2* female mice continued to lose weight at the same rate. Collectively, these data show that, as with body temperature, the beneficial impact of XJB-5-131 on weight loss in *R6/2* animals was sex-dependent, with male mice responding better to drug treatment.

### XJB-5-131 improves performance on the fixed speed rotarod in *R6/2* mice

**Fig 5:**
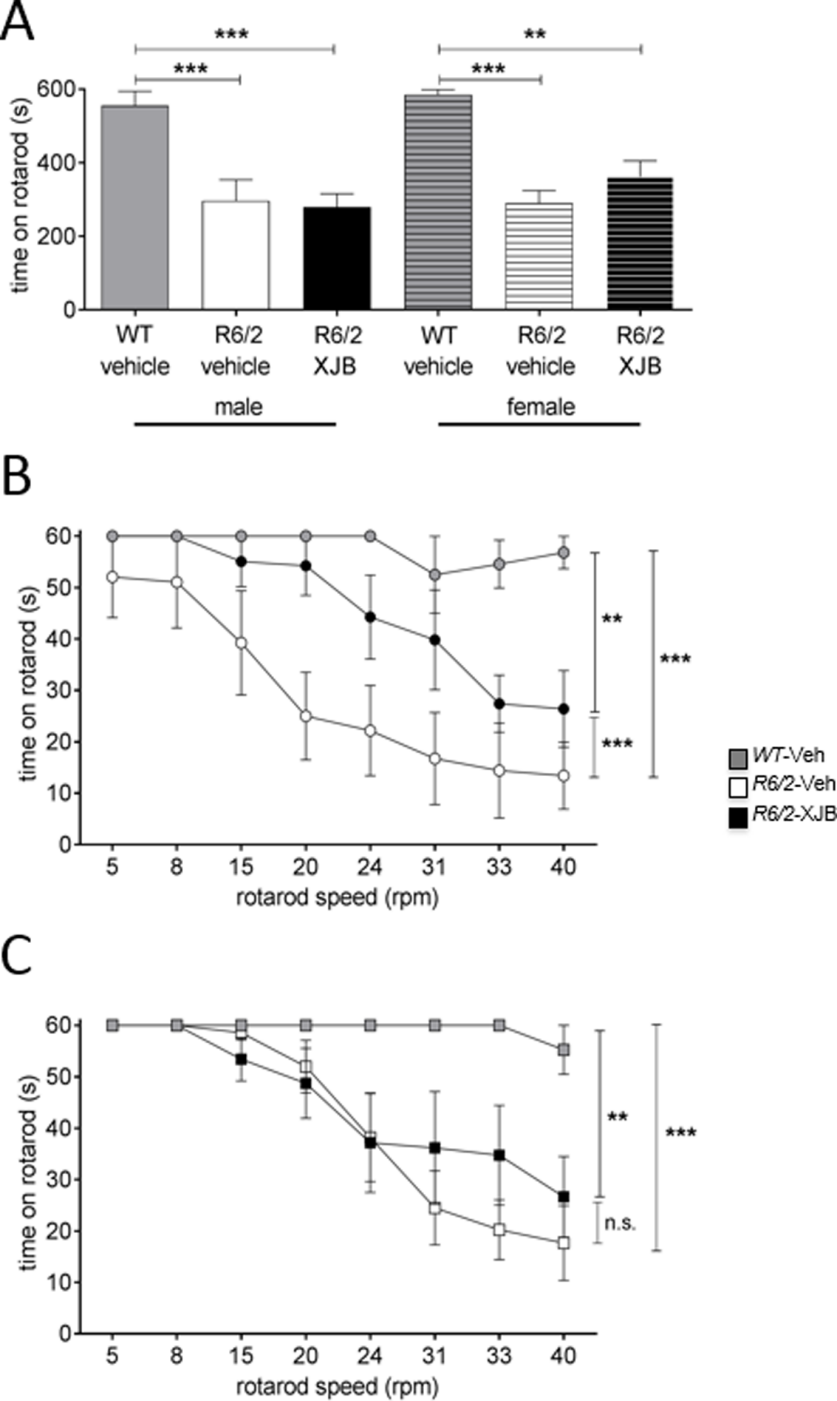
Mice were tested on the accelerating (A) and fixed speed (B, C) rotarod tasks at 12 weeks of age. All *R6/2* mice were impaired at the accelerating rotarod task relative to WT mice (A). *R6/2* male mice showed the same level of impairment regardless of treatment. However, the deficit in XJB-5-131-treated *R6/2* female mice was less than in vehicle-treated mice (A). Both groups of *R6/2* male mice were impaired on the fixed speed rotarod task relative to WT mice (B). However, XJB-5-131-treated male mice performed significantly better than vehicle-treated *R6/2* mice (B). Both groups of *R6/2* female mice were impaired relative to WT female mice (C). There was no difference between XJB-5-131- and vehicle- treated *R6/2* female mice, but the difference between the XJB-5-131-treated mice and WT mice (p<0.01) was less than between vehicle-treated *R6/2* and WT groups (C). ** p<0.01, *** p<0.001. Grey symbols, WT; white symbols, *R6/2*-Veh; black symbols, *R6/2*-XJB. Data are mean ± SEM. Mouse numbers: n = 6 per group.

Mice were tested once at 12 weeks on the accelerating rotarod task to measure motor skill (Fig 5A). *R6/2* male mice were impaired relative to WT mice regardless of treatment (p<0.001 for both XJB-5-131- and *R6/2*-Veh mice). The results for the female mice were similar. *R6/2*-XJB female mice (p<0.01) tended to be smaller than *R6/2*-Veh female mice (p<0.001), but this phenotype did not reach statistical significance (Fig 5A).

Mice were tested on the fixed speed rotarod at 12 weeks of age (Fig 5B and 5C). The *R6/2*-Veh male mice performed poorly relative to WT littermates (p<0.001). However, drug treatment improved the performance of *R6/2*-XJB relative to *R6/2*-Veh male mice at all speeds (Fig 5B); p<0.001). There was little difference in performance between *R6/2*-XJB and *R6/2*-Veh females (Fig 5B). Thus, treatment with XJB-5-131 produced a sex-dependent effect on the fixed speed rotarod task, with improvement observed in male mice.

### XJB-5-131 improves some behaviours of *R6/2* mice in the open field

Mice were assessed in the open field test at 7, 12 and 17 weeks (S1 Table). In *R6/2*-Veh males were not distinguishable from WT at 8 weeks. However, behavioural phenotypes developed at later ages. In *R6/2*-Veh males, the pathlength travelled was shorter than that of WT male mice at 12 weeks (p<0.05) (S1 Table). By 17 weeks, when the *R6/2* phenotype was becoming apparent, *R6/2*-Veh animals reared less frequently against walls and produced less faecal boli relative to WT animals (both comparisons, p<0.001). However, there was no beneficial effect of XJB-5-131 treatment in male animals (S1 Table).

Female *R6/2*-Veh had few behavioural differences relative to WT animals in the open field. Female *R6/2*-XJB mice produced fewer faecal boli than either WT (p<0.001) or *R6/2*-Veh mice (p<0.05), suggesting an XJB-5-131-mediated reduction in anxiety in female mice (S1 Table). Thus, XJB-5-131 produced an improvement in some but not all elements of the phenotype, (S1 Table).

### Treatment with XJB-5-131 temporarily improves SHIRPA scores in *R6/2* mice

**Fig 6:**
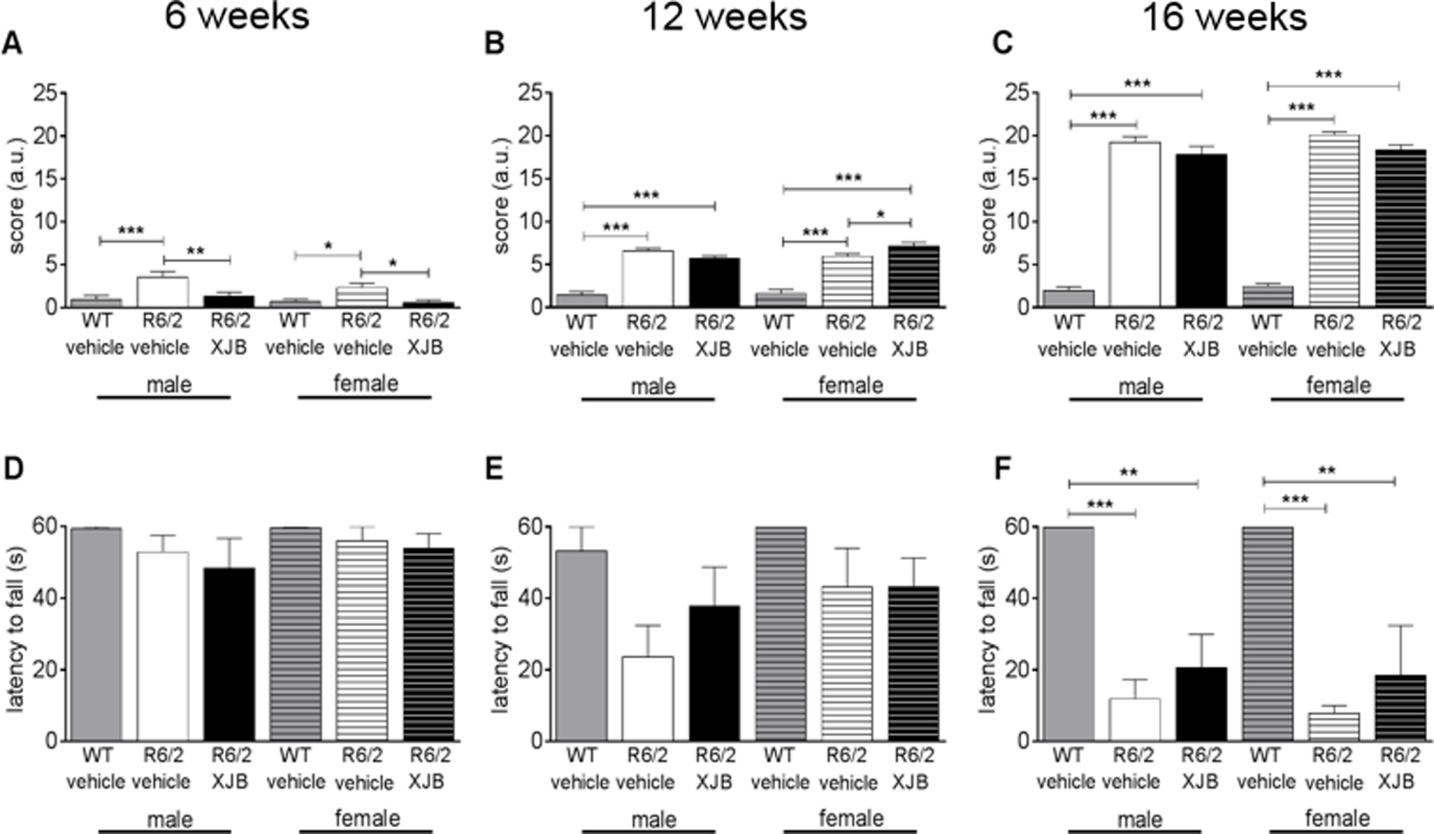
Mice were assessed on the SHIRPA (A, B, C) and hangwire (D, E, F) tests at 6 (A, D), 12 (B, E) and 16 (C, F) weeks of age. At 6 weeks of age, treatment with XJB-5-131 significantly reduced the deficit seen in vehicle-treated *R6/2* mice (A). SHIRPA scores were higher in all *R6/2* mice relative to WT mice at 12 (B) and 16 (C) weeks with no beneficial treatment effect at either age. There were no differences in hangwire scores at 6 (D) or 12 (E) weeks, but all *R6/2* mice had shorter hangwire latencies relative to WT mice at 16 weeks (F). However, the impairment was reduced in XJB-5-131-treated *R6/2* mice of both sexes, suggesting a slight improvement of phenotype (F). * p<0.05, ** p<0.01, *** p<0.001. Data are mean ± SEM. Mouse numbers: 6 weeks, WT male, WT female, *R6/2*-Veh male, *R6/2*-Veh female, n = 8. *R6/2*-XJB male, n = 7; *R6/2*-XJB female, n = 9. 12 weeks, all groups n = 6. 16 weeks, all groups n = 4.

Mice were assessed for a battery of tests to monitor their overall physiological decline at 6, 12, and 16 weeks. SHIRPA is a general measure of physiology derived from the results of a number of behavioural tests. Test results were quantified using an overall global, graded score, where normal behaviour received a score of 0 and the highest scores corresponding to the greatest degree of abnormality and considered as a measure of overall physiological. At 6 weeks, *R6/2*-Veh mice of both sexes had higher SHIRPA scores than WT mice, consistent with deterioration in the disease animal (Fig 6). XJB-5-131 treatment improved the phenotype at 6 weeks in both male and female *R6/2* mice (*R6/2-*XJB, males, p<0.01; females, p<0.05, (Fig 6A), as indicated by the reduction in the SHIRPA score. However, as disease progressed, the beneficial effect of XJB-5-131 was lost in both sexes (Fig 6B and 6C). Grip strength was measured by the hangwire test, where the longer latency to fall indicated an improvement in motor function. Grip strength deteriorated in *R6/2*-Veh mice relative to WT mice by 12 weeks, and continued to decline as disease progressed in both sexes regardless of treatment (Fig 6D and 6E). Thus, XJB-5-131 was beneficial at early ages but the benefit was lost as the disease phenotype became severe.

### XJB-5-131 did not improve behaviour of *R6/2* mice as measured by LABORAS

Mice were placed in the LABORAS activity monitoring system for 48 hours at 8 and 14 weeks for automated assessment of locomotor activity, immobility, grooming, eating, drinking and climbing. LABORAS data were analysed separately from light (7am-7pm) and dark (7pm-7am) phases, since most activity in mice, being nocturnal, takes place during the dark phase.

### Activity in light phase

There were no substantial differences between genotypes detected during the light phase in either sex regardless of treatment (Table 1).

**Table 1:** LABORAS analysis of activity at 8 and 14 weeks. Data are total time at each activity over 48 hours (means ± SEM).

**8 weeks light phase activity**							
	**sex**	**WT-Veh**	***R6/2*-Veh**	***R6/2*-XJB**	**WT-Veh vs *R6/2*-Veh**	**WT-Veh vs *R6/2*-XJB**	***R6/2*-Veh vs *R6/2*-XJB**
					**p value**	**p value**	**p value**
**Climbing (s)**	M	2265 ± 683	1506 ± 336	2507 ± 772	0.984	0.999	0.964
	F	2039 ± 263	3187 ± 367	2147 ± 368	0.881	0.999	0.901
**Locomotion (s)**	M	1341 ± 177	938 ± 118	1204 ± 229	0.403	0.881	0.667
	F	932 ± 90	988 ± 141	954 ± 70	0.962	0.994	0.986
**Immobility (s)**	M	61875 ± 2391	61845 ± 831	61996 ± 1472	0.999	0.999	0.999
	F	64704 ± 1195	60449 ± 1611	61362 ± 1157	0.096	0.225	0.89
**Rearing (s)**	M	724 ± 118	1171 ± 74	1311 ± 158	0.441	0.25	0.92
	F	630 ± 178	1093 ± 301	1202 ± 166	0.631	0.498	0.974
**Grooming (s)**	M	4508 ± 178	4380 ± 369	4236 ± 741	0.989	0.952	0.986
	F	5598 ± 385	5778 ± 607	5437 ± 773	0.978	0.982	0.923
**Drinking (s)**	M	100 ± 60	213 ± 39	113 ± 46	0.563	0.993	0.633
	F	49 ± 14	122 ± 40	119 ± 25	0.654	0.675	0.999
**Eating (s)**	M	2977 ± 443	3149 ± 460	2320 ± 285	0.983	0.776	0.669
	F	1743 ± 289	2171 ± 268	2674 ± 371	0.902	0.619	0.868
**8 weeks dark phase activity**							
	**sex**	**WT-Veh**	***R6/2*-Veh**	***R6/2*-XJB**	**WT-Veh vs *R6/2*-Veh**	**WT-Veh vs *R6/2*-XJB**	***R6/2*-Veh vs *R6/2*-XJB**
					**p value**	**p value**	**p value**
**Climbing (s)**	M	13340 ± 3442	6094 ± 1155	10127 ± 1309	0.012	0.999	0.251
	F	20398 ± 2174	13952 ± 3125	8689 ± 1496	0.029	<0.001	0.086
**Locomotor activity (s)**	M	3119 ± 286	1775 ± 180	2386 ± 271	<0.001	0.06	0.134
	F	2523 ± 184	1888 ± 151	1790 ± 213	0.015	0.005	0.889
**Immobility (s)**	M	33353 ± 4608	48438 ± 1882	42234 ± 2622	0.008	0.055	0.224
	F	29209 ± 1024	42444 ± 934	44173 ± 2108	<0.001	<0.001	0.66
**Rearing (s)**	M	2125 ± 409	2385 ± 321	2891 ± 266	0.75	0.099	0.347
	F	1687 ± 377	1819 ± 488	2473 ± 477	0.963	0.277	0.406
**Grooming (s)**	M	6855 ± 1056	4167 ± 538	4607 ± 585	0.016	0.048	0.879
	F	8644 ± 789	5502 ± 535	5212 ± 608	0.004	0.002	0.944
**Drinking (s)**	M	226 ± 53	438 ± 83	305 ± 138	0.146	0.751	0.455
	F	269 ± 62	266 ± 84	340 ± 84	0.999	0.265	0.252
**Eating (s)**	M	5260 ± 1034	4452 ± 839	3755 ± 722	0.683	0.277	0.751
	F	2357 ± 625	3180 ± 1199	4425 ± 904	0.687	0.109	0.429
**14 weeks light phase activity**							
	**sex**	**WT-Veh**	***R6/2*-Veh**	***R6/2*-XJB**	**WT-Veh vs *R6/2*-Veh**	**WT-Veh vs *R6/2*-XJB**	***R6/2*-Veh vs *R6/2*-XJB**
					**p value**	**p value**	**p value**
**Climbing (s)**	M	1784 ± 784	1262 ± 479	1385 ± 736	0.975	0.985	0.999
	F	1765 ± 707	2561 ± 473	932 ± 261	0.953	0.957	0.834
**Locomotor activity (s)**	M	1302 ± 135	1552 ± 291	965 ± 375	0.917	0.856	0.629
	F	1002 ± 129	1612 ± 717	1587 ± 229	0.754	0.771	0.999
**Immobility (s)**	M	61240 ± 2043	56347 ± 1799	54501 ± 7236	0.742	0.573	0.958
	F	65539 ± 1242	56161 ± 6205	53045 ± 4214	0.249	0.097	0.848
**Rearing (s)**	M	722 ± 111	2521 ± 606	2626 ± 886	0.176	0.103	0.992
	F	803 ± 222	2245 ± 848	1981 ± 752	0.682	0.567	0.981
**Grooming (s)**	M	5593 ± 635	2784 ± 916	4424 ± 1508	0.194	0.735	0.551
	F	5196 ± 285	3600 ± 650	1704 ± 836	0.367	0.018	0.251
**Drinking (s)**	M	55 ± 24	424 ± 210	782 ± 610	0.914	0.708	0.919
	F	72 ± 10	927 ± 571	1185 ± 257	0.457	0.275	0.928
**Eating (s)**	M	2321 ± 553	3620 ± 1098	5316 ± 1998	0.817	0.36	0.711
	F	1213 ± 247	3379 ± 1251	10334 ± 3706	0.73	0.013	0.061
**14 weeks dark phase activity**							
	**sex**	**WT-Veh**	***R6/2*-Veh**	***R6/2*-XJB**	**WT-Veh vs *R6/2*-Veh**	**WT-Veh vs *R6/2*-XJB**	***R6/2*-Veh vs *R6/2*-XJB**
					**p value**	**p value**	**p value**
**Climbing (s)**	M	10374 ± 3678	3398 ± 1346	3048 ± 882	0.025	0.019	0.988
	F	14887 ± 4628	5243 ± 689	3025 ± 1104	0.008	0.002	0.716
**Locomotor activity (s)**	M	3585 ± 478	3106 ± 738	1682 ± 426	0.733	0.02	0.089
	F	3089 ± 305	2833 ± 1118	2306 ± 468	0.951	0.631	0.81
**Immobility (s)**	M	34807 ± 4980	40364 ± 4001	43036 ± 5480	0.682	0.442	0.914
	F	31671 ± 3252	39949 ± 4463	41604 ± 2801	0.332	0.213	0.954
**Rearing (s)**	M	2554 ± 446	3641 ± 629	3263 ± 451	0.512	0.706	0.905
	F	1888 ± 552	3438 ± 1709	3014 ± 1146	0.705	0.522	0.951
**Grooming (s)**	M	6516 ±757	3016 ± 1043	5301 ± 1423	0.088	0.717	0.326
	F	7718 ± 999	3607 ± 1136	2251 ±686	0.006	<0.001	0.48
**Drinking (s)**	M	214 ± 71	1173 ± 644	1799 ± 1284	0.554	0.217	0.774
	F	226 ± 71	1774 ± 942	1902 ± 433	0.097	0.068	0.982
**Eating (s)**	M	4716 ± 913	5268 ± 2045	6507 ± 1770	0.964	0.684	0.832
	F	2230 ± 403	6318 ± 2620	10324 ± 1330	0.342	0.027	0.356

### Activity in dark phase

**Fig 7:**
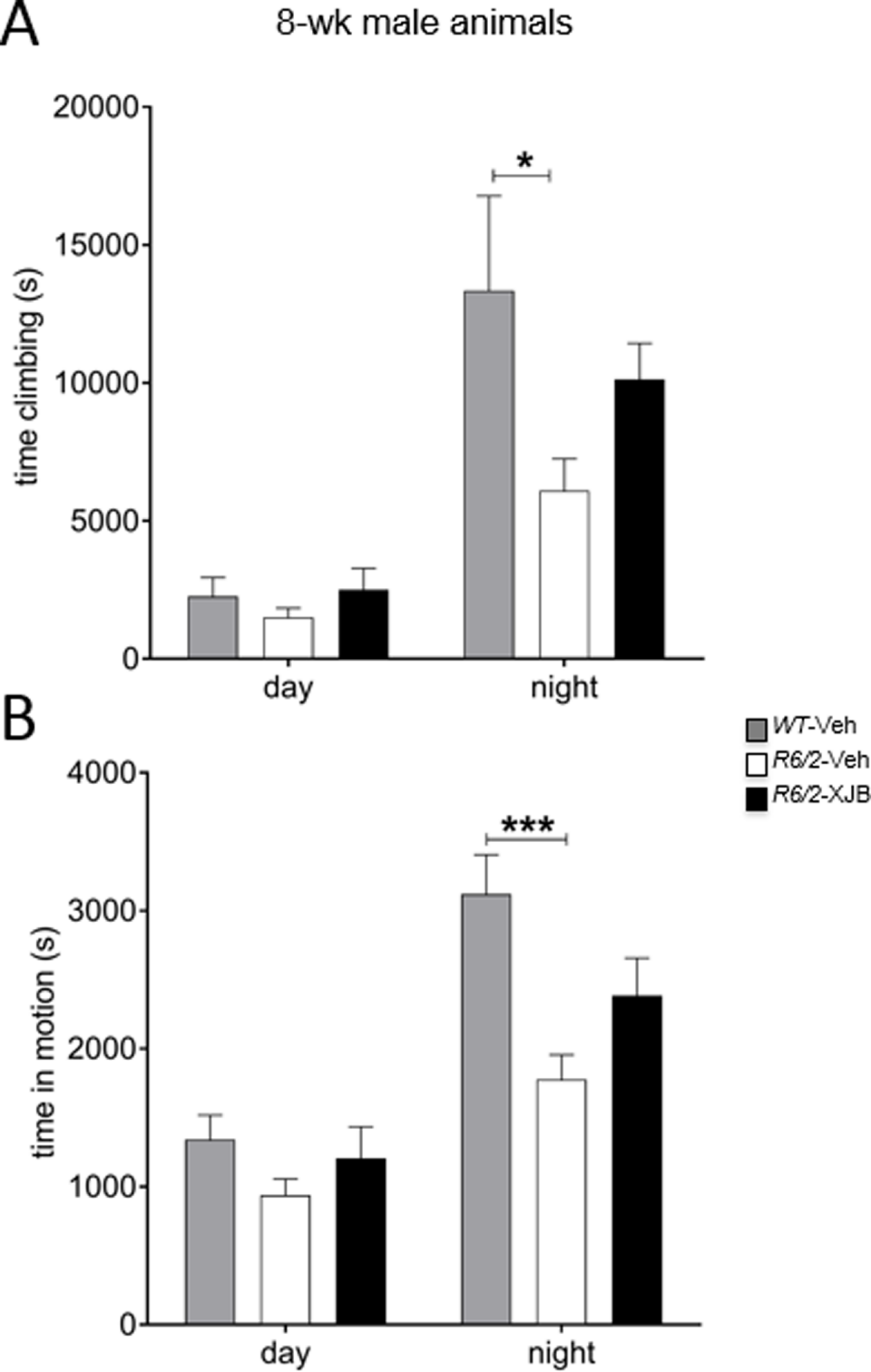
LABORAS analysis of activity at 8 weeks of age. Treatment with XJB-5-131 improved the phenotype in male *R6/2* mice in terms of time climbing (A), and time active (B). * p<0.05, ** p<0.01, *** p<0.001. Grey bars, WT; white bars, *R6/2*-Veh; black bars, *R6/2*-XJB. Data are mean ± SEM. Mouse numbers: WT male, WT female, *R6/2*-Veh male, *R6/2*-Veh female, n = 8. *R6/2*-XJB male, n = 7; *R6/2*-XJB female, n = 9.

A genotype effect was more obvious during the dark phase by 8 weeks of age. In both sexes, WT mice performed better than did *R6/2-*Veh mice with respect to climbing, locomotor activity, immobility and grooming (both comparisons, p<0.05; Table 1) (Fig 7A and 7B). Male and female WT mice continued to perform better at 14 weeks in climbing activity (Table 1), and females groomed more frequently at this age. However, there were no effects of XJB-5-131 treatment in either sex at any age.

### Glycosuria

Several studies have shown that a significant proportion of *R6/2* mice gradually develop increased quantities of sugar in the urine (glycosuria) [32, 33]. Glycosuria was not observed in any WT mice at either 12 or 16 weeks, but is significantly apparent in the R6/2 line, especially at 16 weeks. In *R6/2* at 12 weeks of age, there was no significant difference between Veh and XJB male mice in numbers of mice showing glycosuria (Table 2). However, more *R6/2* female mice were diabetic if XJB compared to Veh treated (p<0.01, Table 2). By 16 weeks, there were no differences in *R6/2* mice between Veh and XJB groups, with all mice showing signs of glycosuria (Table 2).

**Table 2:** Glycosuria at 12 and 16 weeks. Numbers of mice per group showing glycosuria.

	12 weeks (n = 6)	16 weeks (n = 4)
**WT-Veh male**	0	0
**WT-Veh female**	0	0
***R6/2*-Veh male**	1	4
***R6/2*-XJB male**	3	4
***R6/2*-Veh female**	1	4
***R6/2*-XJB female**	5 **	4

** p<0.01, comparison of *R6/2*-Veh and *R6/2*-XJB female mice.

### Survival

**Fig 8:**
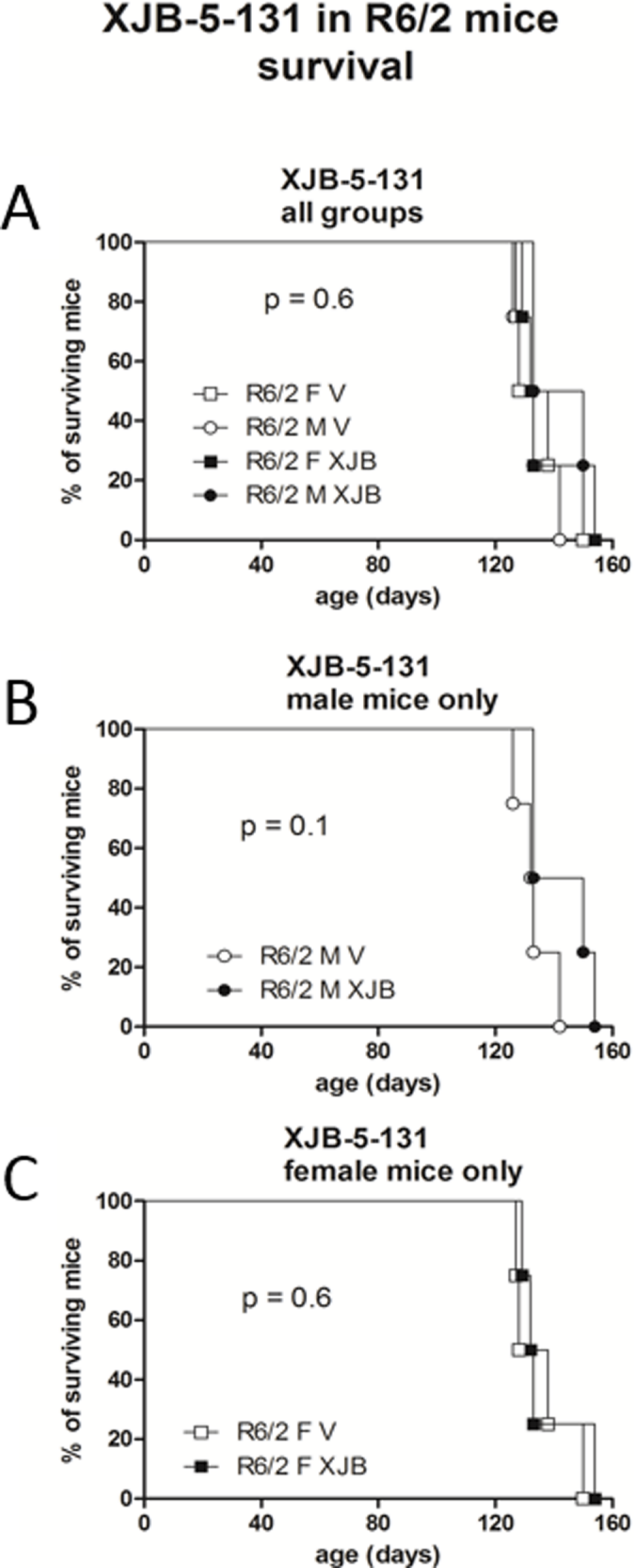
Survival analysis for the *R6/2* mice. Only mice kept beyond the 12 weeks timepoint are represented. There was no effect of XJB-5-131 on age at death in either male (B) or female (C) mice.

From 19 weeks onwards, groups of *R6/2* mice became smaller as mice were killed due to ill-health (Table 3). There were no effects of XJB-5-131 on survival in either male (p = 0.1) or female *R6/2* mice (p = 0.6) (Fig 8), (Table 3). No WT mice died from ill-health during the study.

**Table 3:** The number of mice per group during the experiment.

		number of mice per group					
		WT-Veh male	WT-Veh female	*R6/2*-Veh male	*R6/2*-XJB male	*R6/2*-Veh female	*R6/2*-XJB female
**age (weeks)**	4	8	8	8	7	8	9
	5	8	8	8	7	8	9
	6	8	8	8	7	8	9
	7	8	8	8	7	8	9
	8	8	8	8	7	8	9
	9	6	6	6	6	6	6
	10	6	6	6	6	6	6
	11	6	6	6	6	6	6
	12	6	6	6	6	6	6
	13	4	4	4	4	4	4
	14	4	4	4	4	4	4
	15	4	4	4	4	4	4
	16	4	4	4	4	4	4
	17	4	4	4	4	4	4
	18	4	4	3 *	4	4	4
	19	4	4	1 *	4	2 *	3 *
	20	4	4	0	2 *	2 *	1 *
	21	4	4	0	2 *	1 *	1 *
	22	4	4	0	1 *	0	1 *

Reductions in numbers are where mice were killed for timecourse histology, except where indicated by:

*’ which represents mice killed as a result of ill health.

### Somatic expansion occurs in both male and female *R6/2* animals, and is suppressed by XJB-5-131 in all brain regions measured

**Fig 9:**
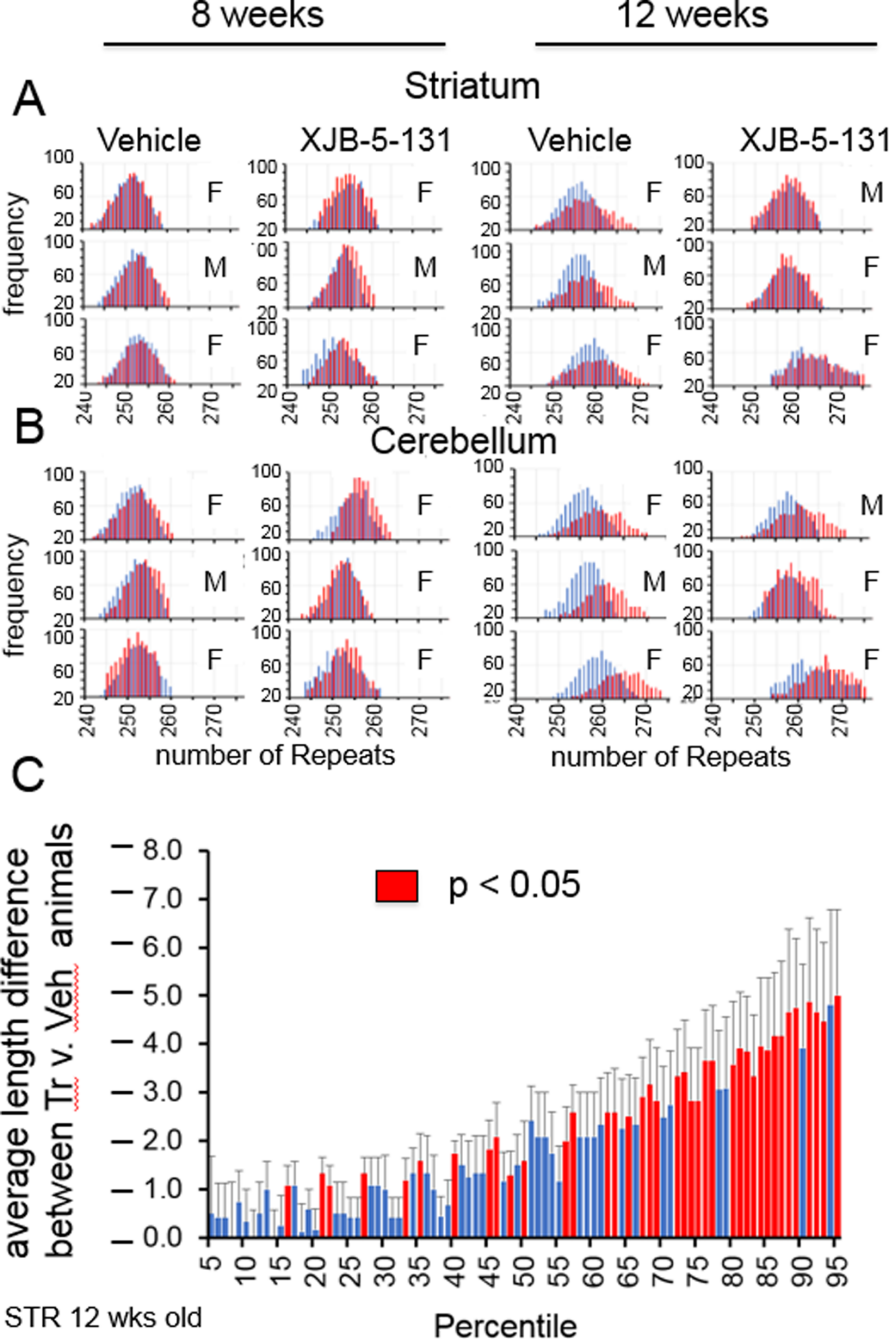
Somatic CAG expansion is suppressed in *R6/2* mice by XJB-5-131 treatment. The CAG repeat lengths in the striatum (A) and cerebellum (B) of *R6/2* mice at 8 or 12 weeks with treatment beginning at 4 weeks. Expansions were observed as a shift in the size distribution of CAG lengths for each mouse (red) compared to the size at weaning (blue). Treatment with XJB-5-131 suppresses expansion in both brain regions. The gender of the mice is designated by ‘M’ (male) or ‘F’ (female). (C) The impact of XJB-5-131 on expansion in the striatum as determined using a quantile analysis [10]. The combined size distribution from the striatum in treated and untreated groups was divided into 99 cells. Shown is the size differences in untreated v. treated striatum along the distribution at 12 weeks. Red are cells in which there was a significant increase in the untreated versus the treated (p < 0.05). Blues are cells with no significant increases. Bracketed lines represent 1 SE (Standard Error of the Mean). The significance is listed for each cell in S2 and S3 Tables. (n = 3 mice per treatment group).

We have shown previously that XJB-5-131 suppresses somatic expansion in *HdHQ(150/150)* mice, and delays the onset of toxicity [10]. Thus, we asked whether somatic expansion was suppressed by XJB-5-131 in early onset *R6/2* mice, which display severe decline and death between 17–22 weeks [17]. We evaluated 14 mice in a blinded study. When unblinded, the results were striking. Of 14 mice evaluated (S1 and S2 Figs), expansions were not observed in any mouse at 8 weeks **(**Fig 9A and 9B), but were prominent at 12 weeks exclusively in *R6/2-*Veh mice (Fig 9A and 9B). Importantly, XJB-5-131 treatment suppressed expansion in both the striatum and in the cerebellum of these 12-week R6/2-XJB mice (Fig 9A and 9B)) Quantile statistics confirmed that expansions were significantly smaller in treated animal, which was obvious at the leading edge of the distribution (Fig 9C),and (S2 and S3 Tables) in both the striatum and in the cerebellum of these animals. The tract lengths were shorter in the treated samples, primarily at the leading edge in both brain regions (Fig 9C), and (S2 and S3 Tables).

### Inclusions are suppressed in *R6/2* mice by XJB-5-131 in brain regions measured

**Fig 10:**
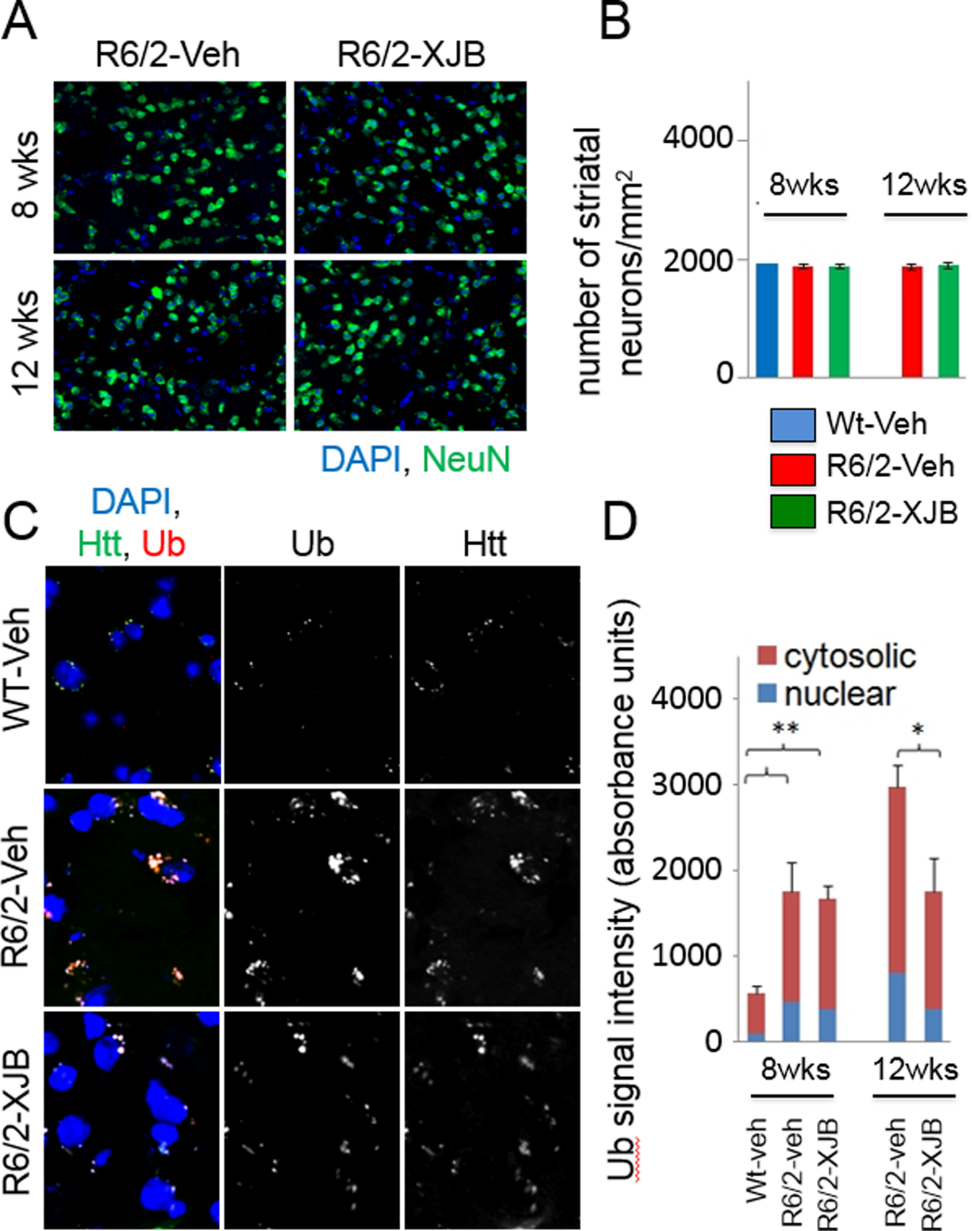
Neuronal loss and inclusion bodies assessed by histology. There was no observable neurodegeneration (loss of neurons = green stained cells; neurons labelled with anti-NeuN antibody) in the striatum of *R6/2* mice by 12 weeks of age (A). Neuronal numbers in the striatum of *R6/2* mice are similar to age-matched WT mice (B). Inclusion bodies (of ubiquitinated protein, labelled with anti-ubiquitin antibody) were increased in *R6/2* mouse striatum compared to matched WT (at 12 weeks). This increase was inhibited by treatment with XJB-5-131 (C). These inclusion bodies are observed in both the nuclear (colocalize with nuclear label = DAPI in (C), blue bars) and the cytosolic (non-nuclear, red bars) compartments (D).

Subsets of mice were evaluated for histology (n = 2 per group) at 8 and 12 weeks (Fig 10). There was no measureable neuronal loss in WT or *R6/2*-Veh mice in either treatment group of either sex at 8 weeks, as expected in this line (Fig 10A and 10B). However, independent of sex, the 12-week *R6/2*-XJB-5-131 mice had fewer inclusion bodies (both nuclear and cytosolic) than *R6/2*-V mice (Fig 10C and 10D). These results suggest that treatment with XJB-5-131 at 2mg/kg in *R6/2* mice supressed somatic expansion and had at least some beneficial effect, as judged by a reduction in a prominent marker for disease progression.

## Discussion

There are no effective therapeutic compounds available that offset the devastating progressive effects of HD. Previously, we have reported that the synthetic radical and electron scavenger, XJB-5-131, attenuates oxidative damage and blocks decline in rotarod performance of *HdhQ(150/150)* mice, if treatment begins before [9] or after the phenotype develops [8]. Here, we asked whether XJB-5-131 can improve the outcome in *R6/2* mice, an HD model with an early onset and severe phenotype. Chronic treatment of *R6/2* mice produced a number of improvements in the phenotype. The strongest effect of the drug on behavioural parameters was seen with the fixed speed rotarod, where *R6/2* XJB-5-131-treated male mice performed significantly better than vehicle-treated *R6/2* mice. Although the effect was weaker in female mice, drug treatment still ameliorated the decline in motor performance on the rotarod. Additional improvements in the phenotype were seen with maintenance of body weight and temperature. The behavioural features in the open field were complex even between genotype activity, and XJB-5-131 did not appear to improve it. When taken together, however, treatment with the mitochondrial-specific compound XJB-5-131 improved several key features in *R6/2* mice. Metabolic deficits are known to be a feature of both HD patients and *R6/2* mice [34, 35]. We did not measure metabolism in the mice in the current study, but the ability of XJB-5-131-treated mice to maintain weight, temperature and muscular strength is indicative of a restoration of the energy imbalance. XJB-5-131 did not extend survival at the dose used. However, the overall benefits in late onset features in *HdhQ(150/150)* mice and in severe, early onset features in *R6/2* mice, taken together, suggest that XJB-5-131 has therapeutic value during the lifetime of these animals.

A second important finding from the analysis was the marked sex differences in response to treatment with XJB-5-131, a result that mirrored results from some of our earlier experiment with *HdhQ(150/150)* [8]. Drug treatment produced stronger beneficial effects in male mice with regard to maintenance of weight, locomotors activity and performance in the fixed speed rotarod task. There were indications with glycosuria testing at 12 weeks, and eating data at 14 weeks, that treatment with XJB-5-131 may have exacerbated elements of the phenotype in female mice. However, female *R6/2* mice treated with XJB-5-131 showed better performance than male drug-treated mice on the accelerating rotarod, indicating that the effect of XJB-5-131 on the *R6/2* phenotype was complex. It is not clear why XJB-5-131 should produce different results in male and female mice. However, we have shown recently that preconditioning *R6/2* mice with the succinate dehydrogenase inhibitor 3-nitropropionic acid also has sex-dependent effects, but in this case the protective effect was more evident in female than male mice [36]. Sex differences in behaviour and response to treatments have also been shown in a range of other mouse models of HD. For example, female YAC128 mice live longer than male mice [37]. In the 140 CAG knock-in model, female mice groomed more frequently, and displayed increased dark phase running, while male mice showed decreased climbing [38]. Male N171-82Q mice perform less well on the rotarod than female mice [39]. Our own work has shown marked differences in the response of male and female *R6/2* mice to environmental enrichment [40]. Taken together, our data suggest that given the obvious sex differences in phenotypically altered behaviour in HD mice, it should not be assumed that any single therapeutic approach or dose will work the same way or to the same extent in both sexes.

We have previously published work showing that suppression of somatic expansion reduces pathophysiology and significantly delays the onset of motor decline in late onset *HdhQ(150/150)* animals [10], and that treatment with XJB-5-131 reduces both the somatic expansion and the disease phenotype of *HdhQ(150/150)* animals [8–10]. Somatic expansion in *R6/2* mice was absent at 8 weeks of age, a time associated with little to no decline in physical parameters or behavioural features of disease. By 12 weeks, however, the CAG expansion in *R6/2* mice of both sexes was supressed by XJB-5-131, as was inclusion formation. Suppression of somatic expansion correlated with the improvement in phenotypes in treated animals. Furthermore, XJB-5-131 also suppressed inclusion formation in the brains of *R6/2* animals, a biomarker for disease progression. The ability of treatment to suppress these features of disease bolsters the idea that treatment has therapeutic value during the lifetime of these animals.

In summary, XJB-5-131 remains a promising compound. In *HdhQ(150/150)* mice, a milder, late onset model of HD, XJB-5-131 suppressed motor decline in animals treated either pre- or post- onset of phenotype. Here, we have shown that chronic treatment of *R6/2* mice with XJB-5-1-131 caused some improvement in the motor deficit and weight loss seen in *R6/2* mice (especially male mice), reduced the loss of body temperature, reduced the number of inclusions and slowed CAG repeat expansion. We suggest that XJB-5-131 is worthy of further study to elucidate the mechanisms that bring about the beneficial effects, and to determine why there were sex differences in the outcomes.

## Supporting information

S1 Fig Raw data for the sizing of CAG repeats from mice tails at time of weaning. The raw data from the electroporation sizing gels for the CAG repeat regions (from GeneMapper). Each sample (mouse) identifier is an alphanumeric code eg. BRM2085t, where ‘M’ indicates the sex (male), and ‘t’ indicates tail DNA. The x axis units are the length of the PCR product (in base pairs). The y axis is signal intensity. The CAG triplet repeat number is calculated as (CAG)n = (PCR size(bp)-122)/3 *1.0425+1.2088. This calculation takes into account the 3’ and 5’ non-repeat portions, and a normalization factor for CAG repeats running in the sizing gels.
(TIF)

S2 Fig Raw data for the sizing of CAG repeats from mouse striatum at indicated ages. The raw data from the electroporation sizing gels for the CAG repeat regions (from GeneMapper). Each sample (mouse) identifier is an alphanumeric code eg. BRM2085t, where ‘M’ indicates the sex (male), and ‘s’ indicates striatum DNA. The x axis units are the length of the PCR product (in base pairs). The y axis is signal intensity. The CAG triplet repeat number is calculated as (CAG)n = (PCR size(bp)-122)/3 *1.0425+1.2088. This calculation takes into account the 3’ and 5’ non-repeat portions, and a normalization factor for CAG repeats running in the sizing gels.
(TIF)

S3 Fig Raw data for the sizing of CAG repeats from mouse cerebellum at indicated ages. The raw data from the electroporation sizing gels for the CAG repeat regions (from GeneMapper). Each sample (mouse) identifier is an alphanumeric code eg. BRM2085t, where ‘M’ indicates the sex (male), and ‘c’ indicates cerebellar DNA. The x axis units are the length of the PCR product (in base pairs). The y axis is signal intensity. The CAG triplet repeat number is calculated as (CAG)n = (PCR size(bp)-122)/3 *1.0425+1.2088. This calculation takes into account the 3’ and 5’ non-repeat portions, and a normalization factor for CAG repeats running in the sizing gels.
(TIF)

S1 Table The impact of XJB-5-131 on the performance of *R6/2* animals in additional open field behaviour at 7, 12 and 17 weeks. Results of Significant difference are shown.* p<0.05, ** p<0.01, *** p<0.001.
(DOCX)

S2 Table Difference in the repeat size distribution in the striatum of the treated and untreated mice (compared as percentiles). (* for p < 0.05) (SE = Standard Error of the Mean).
(DOCX)

S3 Table Difference in the repeat size distribution in the cerebellum of the treated and untreated mice (compared as percentiles). (* for p < 0.05) (SE = Standard Error of the Mean).
(DOCX)
