# Supplementary figures and images for: XJB-5-131-mediated improvement in physiology and behaviour of the R6/2 mouse model of Huntington's disease is age- and sex- dependent
Source: PLoS One. 2018 Apr 9;13(4):e0194580. doi: 10.1371/journal.pone.0194580 (PMC5890981; doi:10.1371/journal.pone.0194580)

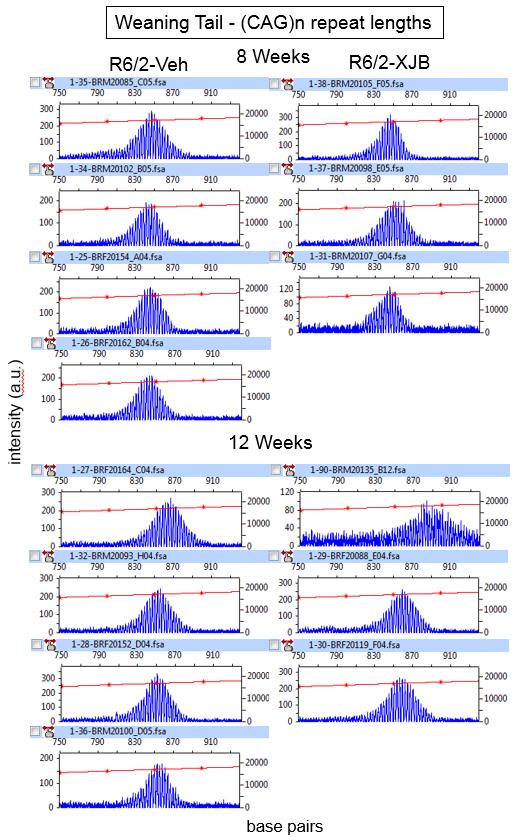

Supplement: S1 Fig — The raw data from the electroporation sizing gels for the CAG repeat regions (from GeneMapper). Each sample (mouse) identifier is an alphanumeric code eg. BRM2085t, where ‘M’ indicates the sex (male), and ‘t’ indicates tail DNA. The x axis units are the length of the PCR product (in base pairs). The y axis is signal intensity. The CAG triplet repeat number is calculated as (CAG)n = (PCR size(bp)-122)/3 *1.0425+1.2088. This calculation takes into account the 3’ and 5’ non-repeat portions, and a normalization factor for CAG repeats running in the sizing gels. (TIF) [file pone.0194580.s001.tif]

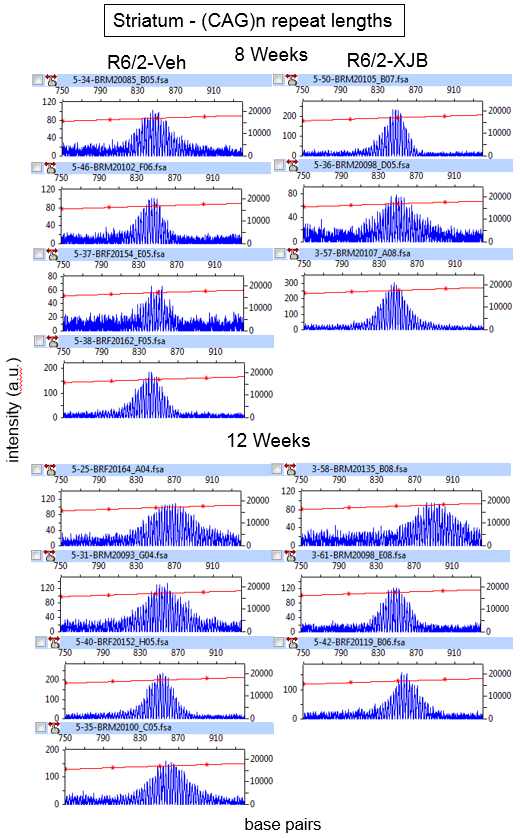

Supplement: S2 Fig — The raw data from the electroporation sizing gels for the CAG repeat regions (from GeneMapper). Each sample (mouse) identifier is an alphanumeric code eg. BRM2085t, where ‘M’ indicates the sex (male), and ‘s’ indicates striatum DNA. The x axis units are the length of the PCR product (in base pairs). The y axis is signal intensity. The CAG triplet repeat number is calculated as (CAG)n = (PCR size(bp)-122)/3 *1.0425+1.2088. This calculation takes into account the 3’ and 5’ non-repeat portions, and a normalization factor for CAG repeats running in the sizing gels. (TIF) [file pone.0194580.s002.tif]

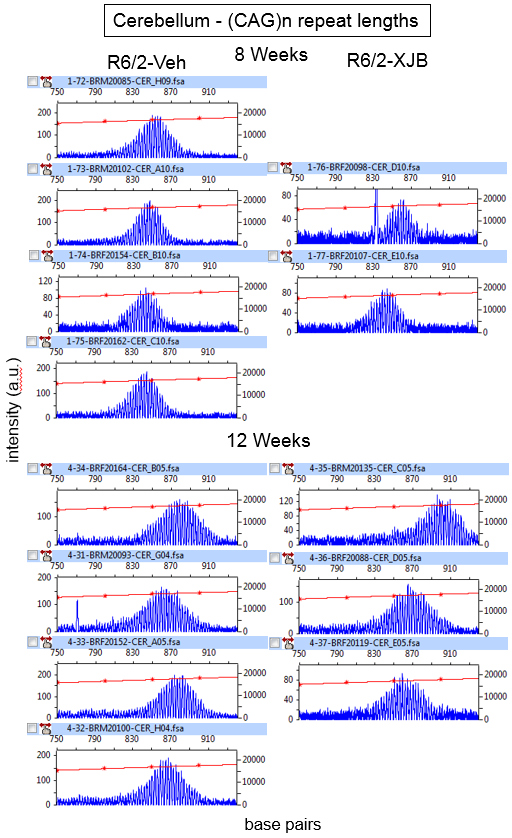

Supplement: S3 Fig — The raw data from the electroporation sizing gels for the CAG repeat regions (from GeneMapper). Each sample (mouse) identifier is an alphanumeric code eg. BRM2085t, where ‘M’ indicates the sex (male), and ‘c’ indicates cerebellar DNA. The x axis units are the length of the PCR product (in base pairs). The y axis is signal intensity. The CAG triplet repeat number is calculated as (CAG)n = (PCR size(bp)-122)/3 *1.0425+1.2088. This calculation takes into account the 3’ and 5’ non-repeat portions, and a normalization factor for CAG repeats running in the sizing gels. (TIF) [file pone.0194580.s003.tif]
