# Supplementary material for: XJB-5-131-mediated improvement in physiology and behaviour of the R6/2 mouse model of Huntington's disease is age- and sex- dependent
Source: PLoS One. 2018 Apr 9;13(4):e0194580. doi: 10.1371/journal.pone.0194580 (PMC5890981; doi:10.1371/journal.pone.0194580)
Supplement: S1 Table — Results of Significant difference are shown.* p<0.05, ** p<0.01, *** p<0.001. (DOCX) [file pone.0194580.s004.docx]

**S1 Table. The impact of XJB-5-131 on the performance of *R6/2* animals in additional open field behaviour at 7, 12 and 17 weeks.** Results of Significant difference are shown.

* p<0.05, ** p<0.01, *** p<0.001

| 7 weeks | | |
| --- | --- | --- |
|  | Male | Female |
| Faecal boli | **-** | **-** |
|  | **-** | *R6/2*-Veh > *R6/2*-XJB * |

| 12 weeks | | |
| --- | --- | --- |
|  | Male | Female |
| Pathlength | WT-Veh > *R6/2*-Veh * | *WT*-Veh > *R6/2*-Veh * |
| Faecal boli | **-** | **-** |

| 17 weeks | | |
| --- | --- | --- |
|  | male | female |
| Rearing supported at wall | WT-Veh > *R6/2*-Veh ** | **-** |
|  |  | **-** |
| Faecal boli | WT-Veh > *R6/2*-Veh *** | WT-Veh > *R6/2*-Veh *** |
|  |  |  |

**Other comparison in males:**  (12 weeks, Fecal boli) WT-Veh > *R6/2*-XJB *; (17 weeks, Rearing) WT-Veh > *R6/2*-XJB*** and (17 weeks, rearing) WT-Veh > *R6/2*-XJB *

**Other comparisons in females:** (8weeks, fecal boli) WT-Veh > *R6/2*-XJB *** and (17 weeks, feacal boli) WT-Veh > *R6/2*-XJB ***; (17 weeks, rearing) WT-Veh > *R6/2*-XJB *
