# Supplementary material for: XJB-5-131-mediated improvement in physiology and behaviour of the R6/2 mouse model of Huntington's disease is age- and sex- dependent
Source: PLoS One. 2018 Apr 9;13(4):e0194580. doi: 10.1371/journal.pone.0194580 (PMC5890981; doi:10.1371/journal.pone.0194580)
Supplement: S2 Table — (* for p < 0.05) (SE = Standard Error of the Mean). (DOCX) [file pone.0194580.s005.docx]

**S2 Table. Difference in the repeat size distribution in the striatum of the treated and untreated mice (compared as percentiles).** (* for p < 0.05) (SE = Standard Error of the Mean).

| percentile | Treatment | | Untreated | | Treated-Untreated Difference | | *P* | |
| --- | --- | --- | --- | --- | --- | --- | --- | --- |
|  |  |  |  |  |  |  |  |  |
|  | Mean | SE | Mean | SE | Mean | SE | Sig | |
|  |  |  |  |  |  |  |  |  |
| 5 perc | 0.000 | 1.155 | 0.500 | 0.289 | -0.500 | 1.190 | 0.692 |  |
| 6 perc | 0.080 | 0.648 | 0.500 | 0.289 | -0.420 | 0.709 | 0.580 |  |
| 7 perc | 0.333 | 0.667 | 0.750 | 0.250 | -0.417 | 0.712 | 0.584 |  |
| 8 perc | 0.000 | 1.155 | 0.000 | 0.000 | 0.000 | 1.155 | 1.000 |  |
| 9 perc | 0.000 | 0.577 | 0.750 | 0.250 | -0.750 | 0.629 | 0.287 |  |
| 10 perc | 0.167 | 0.601 | 0.500 | 0.289 | -0.333 | 0.667 | 0.638 |  |
| 11 perc | 0.000 | 0.577 | 0.000 | 0.000 | 0.000 | 0.577 | 1.000 |  |
| 12 perc | 0.000 | 0.577 | 0.500 | 0.289 | -0.500 | 0.645 | 0.474 |  |
| 13 perc | 0.000 | 0.577 | 1.000 | 0.000 | -1.000 | 0.577 | 0.144 |  |
| 14 perc | 0.000 | 0.577 | 0.000 | 0.000 | 0.000 | 0.577 | 1.000 |  |
| 15 perc | 0.000 | 0.577 | 0.250 | 0.250 | -0.250 | 0.629 | 0.707 |  |
| 16 perc | -0.333 | 0.333 | 0.750 | 0.250 | -1.083 | 0.417 | 0.048 | * |
| 17 perc | -0.090 | 0.501 | 1.000 | 0.000 | -1.090 | 0.501 | 0.082 |  |
| 18 perc | 0.000 | 0.577 | 0.125 | 0.125 | -0.125 | 0.591 | 0.841 |  |
| 19 perc | -0.333 | 0.333 | 0.250 | 0.250 | -0.583 | 0.417 | 0.220 |  |
| 20 perc | 0.333 | 0.333 | 0.500 | 0.289 | -0.167 | 0.441 | 0.721 |  |
| 21 perc | -0.333 | 0.333 | 1.000 | 0.000 | -1.333 | 0.333 | 0.010 | * |
| 22 perc | -0.333 | 0.333 | 0.750 | 0.250 | -1.083 | 0.417 | 0.048 | * |
| 23 perc | 0.000 | 0.577 | 0.500 | 0.289 | -0.500 | 0.645 | 0.474 |  |
| 24 perc | 0.000 | 0.577 | 0.500 | 0.289 | -0.500 | 0.645 | 0.474 |  |
| 25 perc | 0.333 | 0.333 | 0.750 | 0.250 | -0.417 | 0.417 | 0.363 |  |
| 26 perc | 0.333 | 0.333 | 0.750 | 0.250 | -0.417 | 0.417 | 0.363 |  |
| 27 perc | -0.333 | 0.333 | 1.000 | 0.000 | -1.333 | 0.333 | 0.010 | * |
| 28 perc | -0.333 | 0.333 | 0.750 | 0.479 | -1.083 | 0.583 | 0.122 |  |
| 29 perc | -0.333 | 0.333 | 0.750 | 0.479 | -1.083 | 0.583 | 0.122 |  |
| 30 perc | 0.000 | 0.577 | 1.000 | 0.408 | -1.000 | 0.707 | 0.216 |  |
| 31 perc | 0.333 | 0.333 | 0.750 | 0.250 | -0.417 | 0.417 | 0.363 |  |
| 32 perc | 0.333 | 0.333 | 0.750 | 0.250 | -0.417 | 0.417 | 0.363 |  |
| 33 perc | -0.187 | 0.187 | 1.000 | 0.408 | -1.187 | 0.449 | 0.046 | * |
| 34 perc | -0.333 | 0.333 | 1.000 | 0.408 | -1.333 | 0.527 | 0.053 |  |
| 35 perc | -0.333 | 0.333 | 1.250 | 0.479 | -1.583 | 0.583 | 0.042 | * |
| 36 perc | -0.333 | 0.667 | 1.000 | 0.408 | -1.333 | 0.782 | 0.149 |  |
| 37 perc | 0.000 | 0.577 | 1.000 | 0.408 | -1.000 | 0.707 | 0.216 |  |
| 38 perc | 0.333 | 0.333 | 0.775 | 0.259 | -0.442 | 0.422 | 0.344 |  |
| 39 perc | 0.333 | 0.333 | 1.000 | 0.408 | -0.667 | 0.527 | 0.262 |  |
| 40 perc | 0.000 | 0.000 | 1.750 | 0.250 | -1.750 | 0.250 | 0.001 | * |
| 41 perc | 0.000 | 0.577 | 1.500 | 0.289 | -1.500 | 0.645 | 0.068 |  |
| 42 perc | 0.000 | 0.577 | 1.250 | 0.479 | -1.250 | 0.750 | 0.156 |  |
| 43 perc | -0.333 | 0.667 | 1.000 | 0.408 | -1.333 | 0.782 | 0.149 |  |
| 44 perc | -0.333 | 0.667 | 1.000 | 0.408 | -1.333 | 0.782 | 0.149 |  |
| 45 perc | -0.333 | 0.333 | 1.500 | 0.500 | -1.833 | 0.601 | 0.028 | * |
| 46 perc | -0.333 | 0.333 | 1.750 | 0.629 | -2.083 | 0.712 | 0.033 | * |
| 47 perc | 0.333 | 0.333 | 1.500 | 0.500 | -1.167 | 0.601 | 0.110 |  |
| 48 perc | 0.213 | 0.407 | 1.500 | 0.289 | -1.287 | 0.499 | 0.049 | * |
| 49 perc | 0.000 | 0.577 | 1.500 | 0.289 | -1.500 | 0.645 | 0.068 |  |
| 50 perc | -0.333 | 0.667 | 1.250 | 0.479 | -1.583 | 0.821 | 0.112 |  |
| 51 perc | -0.667 | 0.333 | 1.750 | 0.629 | -2.417 | 0.712 | 0.019 | * |
| 52 perc | -0.333 | 0.667 | 1.750 | 0.629 | -2.083 | 0.917 | 0.072 |  |
| 53 perc | -0.333 | 0.667 | 1.750 | 0.629 | -2.083 | 0.917 | 0.072 |  |
| 54 perc | 0.000 | 0.577 | 1.750 | 0.629 | -1.750 | 0.854 | 0.096 |  |
| 55 perc | 0.333 | 0.333 | 1.500 | 0.645 | -1.167 | 0.726 | 0.169 |  |
| 56 perc | 0.000 | 0.577 | 2.000 | 0.408 | -2.000 | 0.707 | 0.037 | * |
| 57 perc | -0.333 | 0.333 | 2.250 | 0.479 | -2.583 | 0.583 | 0.007 | * |
| 58 perc | -0.333 | 0.667 | 1.750 | 0.629 | -2.083 | 0.917 | 0.072 |  |
| 59 perc | -0.333 | 0.667 | 1.750 | 0.629 | -2.083 | 0.917 | 0.072 |  |
| 60 perc | -0.333 | 0.667 | 1.750 | 0.629 | -2.083 | 0.917 | 0.072 |  |
| 61 perc | -0.333 | 0.667 | 2.000 | 0.707 | -2.333 | 0.972 | 0.062 |  |
| 62 perc | -0.333 | 0.333 | 2.250 | 0.750 | -2.583 | 0.821 | 0.025 | * |
| 63 perc | -0.333 | 0.333 | 2.250 | 0.854 | -2.583 | 0.917 | 0.037 | * |
| 64 perc | 0.000 | 0.577 | 2.250 | 0.854 | -2.250 | 1.031 | 0.081 |  |
| 65 perc | -0.333 | 0.667 | 2.175 | 0.545 | -2.508 | 0.861 | 0.033 | * |
| 66 perc | -0.333 | 0.667 | 2.000 | 0.707 | -2.333 | 0.972 | 0.062 |  |
| 67 perc | -0.667 | 0.333 | 2.250 | 0.750 | -2.917 | 0.821 | 0.016 | * |
| 68 perc | -0.667 | 0.333 | 2.500 | 0.866 | -3.167 | 0.928 | 0.019 | * |
| 69 perc | -0.333 | 0.667 | 2.500 | 0.866 | -2.833 | 1.093 | 0.049 | * |
| 70 perc | -0.233 | 0.623 | 2.250 | 0.854 | -2.483 | 1.057 | 0.066 |  |
| 71 perc | 0.000 | 0.577 | 2.750 | 0.946 | -2.750 | 1.109 | 0.056 |  |
| 72 perc | -0.333 | 0.667 | 3.000 | 0.707 | -3.333 | 0.972 | 0.019 | * |
| 73 perc | -0.667 | 0.333 | 2.750 | 1.031 | -3.417 | 1.083 | 0.025 | * |
| 74 perc | -0.333 | 0.667 | 2.500 | 0.866 | -2.833 | 1.093 | 0.049 | * |
| 75 perc | -0.333 | 0.667 | 2.500 | 0.866 | -2.833 | 1.093 | 0.049 | * |
| 76 perc | -0.667 | 0.333 | 3.000 | 1.000 | -3.667 | 1.054 | 0.018 | * |
| 77 perc | -0.667 | 0.333 | 3.000 | 1.080 | -3.667 | 1.130 | 0.023 | * |
| 78 perc | -0.060 | 0.580 | 3.000 | 1.080 | -3.060 | 1.226 | 0.055 |  |
| 79 perc | -0.333 | 0.667 | 2.755 | 1.310 | -3.088 | 1.470 | 0.090 |  |
| 80 perc | -0.333 | 0.667 | 3.250 | 1.109 | -3.583 | 1.294 | 0.039 | * |
| 81 perc | -0.667 | 0.333 | 3.250 | 1.109 | -3.917 | 1.158 | 0.020 | * |
| 82 perc | -0.747 | 0.380 | 3.100 | 1.085 | -3.847 | 1.149 | 0.020 | * |
| 83 perc | -0.333 | 0.667 | 3.000 | 1.080 | -3.333 | 1.269 | 0.047 | * |
| 84 perc | -0.547 | 0.795 | 3.410 | 1.181 | -3.957 | 1.424 | 0.039 | * |
| 85 perc | -0.433 | 0.567 | 3.438 | 1.386 | -3.871 | 1.497 | 0.049 | * |
| 86 perc | -0.667 | 0.333 | 3.500 | 1.258 | -4.167 | 1.302 | 0.024 | * |
| 87 perc | -0.667 | 0.882 | 3.500 | 1.258 | -4.167 | 1.537 | 0.042 | * |
| 88 perc | -0.667 | 0.882 | 4.000 | 1.472 | -4.667 | 1.716 | 0.042 | * |
| 89 perc | -1.000 | 0.577 | 3.750 | 1.315 | -4.750 | 1.436 | 0.021 | * |
| 90 perc | -0.667 | 0.882 | 3.250 | 1.493 | -3.917 | 1.734 | 0.073 |  |
| 91 perc | -0.667 | 0.882 | 4.215 | 1.488 | -4.882 | 1.729 | 0.037 | * |
| 92 perc | -0.667 | 0.882 | 4.000 | 1.472 | -4.667 | 1.716 | 0.042 | * |
| 93 perc | -0.667 | 0.333 | 3.797 | 1.599 | -4.464 | 1.633 | 0.041 | * |
| 94 perc | -1.000 | 1.000 | 3.810 | 1.706 | -4.810 | 1.977 | 0.059 |  |
| 95 perc | -1.000 | 1.000 | 4.000 | 1.472 | -5.000 | 1.780 | 0.038 | * |
