# Supplementary material for: XJB-5-131-mediated improvement in physiology and behaviour of the R6/2 mouse model of Huntington's disease is age- and sex- dependent
Source: PLoS One. 2018 Apr 9;13(4):e0194580. doi: 10.1371/journal.pone.0194580 (PMC5890981; doi:10.1371/journal.pone.0194580)
Supplement: S3 Table — (* for p < 0.05) (SE = Standard Error of the Mean). (DOCX) [file pone.0194580.s006.docx]

**S3 Table. Difference in the repeat size distribution in the cerebellum of the treated and untreated mice (compared as percentiles).** (* for p < 0.05) (SE = Standard Error of the Mean).

| percentile | Treatment | | Untreated | | Treated-Untreated Difference | | *P* | |
| --- | --- | --- | --- | --- | --- | --- | --- | --- |
|  |  |  |  |  |  |  |  |  |
|  | Mean | SE | Mean | SE | Mean | SE | Sig | |
|  |  |  |  |  |  |  |  |  |
| 5 perc | 0.667 | 0.333 | 2.000 | 2.273 | -1.333 | 2.297 | 0.587 |  |
| 6 perc | 1.080 | 0.510 | 1.750 | 2.056 | -0.670 | 2.119 | 0.765 |  |
| 7 perc | 1.333 | 0.333 | 2.377 | 2.247 | -1.044 | 2.272 | 0.665 |  |
| 8 perc | 1.000 | 0.000 | 2.000 | 1.915 | -1.000 | 1.915 | 0.624 |  |
| 9 perc | 1.000 | 0.000 | 2.343 | 2.091 | -1.343 | 2.091 | 0.549 |  |
| 10 perc | 1.000 | 0.000 | 2.500 | 2.021 | -1.500 | 2.021 | 0.491 |  |
| 11 perc | 1.333 | 0.667 | 2.250 | 1.797 | -0.917 | 1.917 | 0.653 |  |
| 12 perc | 1.000 | 0.000 | 3.000 | 1.915 | -2.000 | 1.915 | 0.344 |  |
| 13 perc | 1.000 | 0.000 | 3.000 | 1.915 | -2.000 | 1.915 | 0.344 |  |
| 14 perc | 1.333 | 0.333 | 2.250 | 1.797 | -0.917 | 1.828 | 0.637 |  |
| 15 perc | 1.333 | 0.333 | 3.000 | 1.915 | -1.667 | 1.944 | 0.430 |  |
| 16 perc | 1.000 | 0.000 | 3.220 | 1.810 | -2.220 | 1.810 | 0.275 |  |
| 17 perc | 1.000 | 0.000 | 3.500 | 1.658 | -2.500 | 1.658 | 0.192 |  |
| 18 perc | 1.667 | 0.333 | 2.625 | 1.599 | -0.958 | 1.634 | 0.583 |  |
| 19 perc | 1.333 | 0.333 | 3.250 | 1.797 | -1.917 | 1.828 | 0.342 |  |
| 20 perc | 1.333 | 0.333 | 3.350 | 1.739 | -2.017 | 1.770 | 0.306 |  |
| 21 perc | 1.400 | 0.306 | 3.500 | 1.658 | -2.100 | 1.686 | 0.268 |  |
| 22 perc | 1.667 | 0.333 | 3.750 | 1.377 | -2.083 | 1.417 | 0.201 |  |
| 23 perc | 1.667 | 0.333 | 3.328 | 1.438 | -1.661 | 1.476 | 0.312 |  |
| 24 perc | 1.333 | 0.333 | 3.500 | 1.658 | -2.167 | 1.691 | 0.256 |  |
| 25 perc | 2.000 | 0.577 | 3.750 | 1.548 | -1.750 | 1.652 | 0.338 |  |
| 26 perc | 2.000 | 0.577 | 4.000 | 1.581 | -2.000 | 1.683 | 0.288 |  |
| 27 perc | 1.333 | 0.667 | 4.250 | 1.436 | -2.917 | 1.583 | 0.125 |  |
| 28 perc | 1.667 | 0.333 | 3.750 | 1.181 | -2.083 | 1.228 | 0.150 |  |
| 29 perc | 1.667 | 0.333 | 4.000 | 1.472 | -2.333 | 1.509 | 0.183 |  |
| 30 perc | 2.000 | 0.577 | 4.000 | 1.472 | -2.000 | 1.581 | 0.262 |  |
| 31 perc | 2.000 | 0.577 | 4.250 | 1.436 | -2.250 | 1.548 | 0.206 |  |
| 32 perc | 2.000 | 0.577 | 4.250 | 1.436 | -2.250 | 1.548 | 0.206 |  |
| 33 perc | 1.480 | 0.520 | 4.250 | 1.436 | -2.770 | 1.527 | 0.129 |  |
| 34 perc | 1.667 | 0.333 | 4.500 | 1.555 | -2.833 | 1.590 | 0.135 |  |
| 35 perc | 2.333 | 0.667 | 4.250 | 1.315 | -1.917 | 1.474 | 0.250 |  |
| 36 perc | 2.000 | 0.577 | 4.500 | 1.323 | -2.500 | 1.443 | 0.144 |  |
| 37 perc | 2.000 | 0.577 | 4.500 | 1.323 | -2.500 | 1.443 | 0.144 |  |
| 38 perc | 2.000 | 0.577 | 4.275 | 1.423 | -2.275 | 1.536 | 0.199 |  |
| 39 perc | 2.330 | 0.665 | 4.500 | 1.323 | -2.170 | 1.481 | 0.203 |  |
| 40 perc | 2.000 | 0.577 | 4.750 | 1.548 | -2.750 | 1.652 | 0.157 |  |
| 41 perc | 2.207 | 0.793 | 5.000 | 1.414 | -2.793 | 1.622 | 0.146 |  |
| 42 perc | 2.333 | 0.667 | 4.750 | 1.181 | -2.417 | 1.357 | 0.135 |  |
| 43 perc | 2.000 | 0.577 | 4.500 | 1.323 | -2.500 | 1.443 | 0.144 |  |
| 44 perc | 2.333 | 0.667 | 4.500 | 1.323 | -2.167 | 1.481 | 0.203 |  |
| 45 perc | 2.000 | 0.577 | 4.913 | 1.136 | -2.913 | 1.274 | 0.071 |  |
| 46 perc | 2.000 | 0.577 | 5.250 | 1.315 | -3.250 | 1.436 | 0.073 |  |
| 47 perc | 2.333 | 0.667 | 5.250 | 1.436 | -2.917 | 1.583 | 0.125 |  |
| 48 perc | 2.213 | 0.787 | 5.000 | 1.414 | -2.787 | 1.618 | 0.146 |  |
| 49 perc | 2.333 | 0.667 | 5.000 | 1.414 | -2.667 | 1.563 | 0.149 |  |
| 50 perc | 2.333 | 0.667 | 4.500 | 1.323 | -2.167 | 1.481 | 0.203 |  |
| 51 perc | 2.000 | 0.577 | 5.000 | 1.080 | -3.000 | 1.225 | 0.058 |  |
| 52 perc | 2.000 | 0.577 | 5.000 | 1.080 | -3.000 | 1.225 | 0.058 |  |
| 53 perc | 2.000 | 0.577 | 5.500 | 1.323 | -3.500 | 1.443 | 0.060 |  |
| 54 perc | 2.333 | 0.667 | 5.500 | 1.323 | -3.167 | 1.481 | 0.086 |  |
| 55 perc | 2.333 | 0.667 | 5.250 | 1.315 | -2.917 | 1.474 | 0.105 |  |
| 56 perc | 2.667 | 0.882 | 5.250 | 1.315 | -2.583 | 1.583 | 0.164 |  |
| 57 perc | 2.333 | 0.882 | 5.500 | 1.190 | -3.167 | 1.481 | 0.086 |  |
| 58 perc | 2.000 | 0.577 | 5.200 | 1.098 | -3.200 | 1.241 | 0.050 |  |
| 59 perc | 2.000 | 0.577 | 5.500 | 1.323 | -3.500 | 1.443 | 0.060 |  |
| 60 perc | 2.333 | 0.667 | 5.500 | 1.323 | -3.167 | 1.481 | 0.086 |  |
| 61 perc | 2.667 | 0.882 | 5.750 | 1.181 | -3.083 | 1.474 | 0.091 |  |
| 62 perc | 2.333 | 0.882 | 5.750 | 1.181 | -3.417 | 1.474 | 0.068 |  |
| 63 perc | 2.333 | 0.882 | 5.750 | 1.109 | -3.417 | 1.417 | 0.061 |  |
| 64 perc | 2.333 | 0.882 | 6.000 | 1.080 | -3.667 | 1.394 | 0.047 | * |
| 65 perc | 2.000 | 0.577 | 5.925 | 1.344 | -3.925 | 1.463 | 0.044 | * |
| 66 perc | 2.667 | 0.882 | 5.750 | 1.181 | -3.083 | 1.474 | 0.091 |  |
| 67 perc | 2.333 | 0.882 | 5.750 | 1.181 | -3.417 | 1.474 | 0.068 |  |
| 68 perc | 2.333 | 0.882 | 5.750 | 1.181 | -3.417 | 1.474 | 0.068 |  |
| 69 perc | 2.667 | 0.667 | 6.250 | 1.109 | -3.583 | 1.294 | 0.039 | * |
| 70 perc | 2.567 | 1.105 | 6.000 | 1.080 | -3.433 | 1.545 | 0.077 |  |
| 71 perc | 2.667 | 1.202 | 6.250 | 0.946 | -3.583 | 1.530 | 0.066 |  |
| 72 perc | 2.667 | 0.882 | 6.500 | 1.190 | -3.833 | 1.481 | 0.049 | * |
| 73 perc | 2.333 | 0.882 | 6.000 | 1.080 | -3.667 | 1.394 | 0.047 | * |
| 74 perc | 2.333 | 0.882 | 6.000 | 1.080 | -3.667 | 1.394 | 0.047 | * |
| 75 perc | 2.917 | 0.917 | 6.500 | 0.957 | -3.583 | 1.325 | 0.043 | * |
| 76 perc | 2.667 | 1.202 | 6.500 | 0.957 | -3.833 | 1.537 | 0.055 |  |
| 77 perc | 2.333 | 1.333 | 6.250 | 0.946 | -3.917 | 1.635 | 0.062 |  |
| 78 perc | 2.333 | 1.333 | 6.500 | 1.190 | -4.167 | 1.787 | 0.067 |  |
| 79 perc | 2.333 | 0.882 | 6.505 | 0.871 | -4.172 | 1.239 | 0.020 | * |
| 80 perc | 2.667 | 1.202 | 6.750 | 0.854 | -4.083 | 1.474 | 0.039 | * |
| 81 perc | 2.333 | 1.333 | 6.750 | 0.854 | -4.417 | 1.583 | 0.038 | * |
| 82 perc | 2.587 | 1.226 | 6.350 | 0.943 | -3.763 | 1.547 | 0.059 |  |
| 83 perc | 2.333 | 1.333 | 6.700 | 1.060 | -4.367 | 1.703 | 0.050 |  |
| 84 perc | 2.787 | 1.319 | 7.250 | 0.946 | -4.463 | 1.623 | 0.040 | * |
| 85 perc | 2.567 | 1.233 | 6.812 | 0.812 | -4.246 | 1.477 | 0.035 | * |
| 86 perc | 2.333 | 1.333 | 6.750 | 0.854 | -4.417 | 1.583 | 0.038 | * |
| 87 perc | 2.223 | 1.747 | 7.250 | 0.854 | -5.027 | 1.945 | 0.049 | * |
| 88 perc | 2.667 | 1.667 | 7.450 | 0.818 | -4.783 | 1.857 | 0.050 |  |
| 89 perc | 2.333 | 1.333 | 7.250 | 0.946 | -4.917 | 1.635 | 0.030 | * |
| 90 perc | 2.333 | 1.333 | 7.000 | 0.816 | -4.667 | 1.563 | 0.031 | * |
| 91 perc | 2.333 | 1.856 | 7.500 | 0.645 | -5.167 | 1.965 | 0.047 | * |
| 92 perc | 2.333 | 1.856 | 7.500 | 0.866 | -5.167 | 2.048 | 0.053 |  |
| 93 perc | 2.667 | 1.667 | 7.420 | 0.700 | -4.753 | 1.808 | 0.047 | * |
| 94 perc | 2.333 | 1.856 | 7.500 | 0.645 | -5.167 | 1.965 | 0.047 | * |
| 95 perc | 2.333 | 1.856 | 7.500 | 0.866 | -5.167 | 2.048 | 0.053 |  |
